# A Quantitative Molecular Orbital Perspective of the Chalcogen Bond

**DOI:** 10.1002/open.202000323

**Published:** 2021-02-17

**Authors:** Lucas de Azevedo Santos, Stephanie C. C. van der Lubbe, Trevor A. Hamlin, Teodorico C. Ramalho, F. Matthias Bickelhaupt

**Affiliations:** ^1^ Department of Theoretical Chemistry Amsterdam Institute for Molecular and Life Sciences (AIMMS), Amsterdam Center for Multiscale Modeling (ACMM) Vrije Universiteit Amsterdam De Boelelaan 1083 1081 HV Amsterdam The Netherlands; ^2^ Department of Chemistry Institute of Natural Sciences Federal University of Lavras CEP 37200-900 Lavras-MG Brazil; ^3^ Center for Basic and Applied Research University Hradec Kralove Hradec Kralove Czech Republic; ^4^ Institute for Molecules and Materials Radboud University Nijmegen Heyendaalseweg 135 6525 AJ Nijmegen The Netherlands

**Keywords:** activation strain model, chalcogen bonding, density functional calculations, energy decomposition analysis, noncovalent interactions

## Abstract

We have quantum chemically analyzed the structure and stability of archetypal chalcogen‐bonded model complexes D_2_Ch⋅⋅⋅A^−^ (Ch = O, S, Se, Te; D, A = F, Cl, Br) using relativistic density functional theory at ZORA‐M06/QZ4P. Our purpose is twofold: (i) to compute accurate trends in chalcogen‐bond strength based on a set of consistent data; and (ii) to rationalize these trends in terms of detailed analyses of the bonding mechanism based on quantitative Kohn‐Sham molecular orbital (KS‐MO) theory in combination with a canonical energy decomposition analysis (EDA). At odds with the commonly accepted view of chalcogen bonding as a predominantly electrostatic phenomenon, we find that chalcogen bonds, just as hydrogen and halogen bonds, have a significant covalent character stemming from strong HOMO−LUMO interactions. Besides providing significantly to the bond strength, these orbital interactions are also manifested by the structural distortions they induce as well as the associated charge transfer from A^−^ to D_2_Ch.

## Graphical abstract

**Covalency strikes back**! Our quantitative molecular orbital analyses show that chalcogen bonds have a considerable covalent component and are far from being purely electrostatic phenomena. They closely resemble halogen bonds and have similarity with hydrogen bonds.

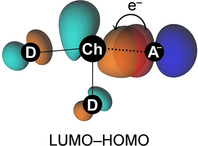

## 1 Introduction

The chalcogen‐bond (ChB) is the net‐attractive intermolecular interaction, often referred to as noncovalent interaction, between a Lewis‐basic chalcogen‐bond acceptor A and a Lewis‐acidic chalcogen‐bond donor D_2_Ch featuring a chalcogen (group 16) atom Ch to which A binds.^[1]^ Nearly 40 years ago, the first systematic study appeared of the chalcogen bond in which S⋅⋅⋅Y (*e*. *g*., Y = S, O, F, Cl, or Br) nonbonded atomic contacts were investigated.^[2]^ Early studies generally characterized chalcogen bonds as being predominantly electrostatic in nature.^[3]^ Later on, the significance of charge transfer from the occupied orbital of a Lewis base into an empty σ*‐type orbital of a chalcogen molecule controlling the chalcogen bond strength was recognized.^[4]^ Chalcogen‐bonding has since found applications in various fields of chemistry,^[1]^ including, supramolecular,^[5]^ biochemistry,^[6]^ spectroscopy^[7]^ and catalysis.^[8]^

**Scheme 1:**
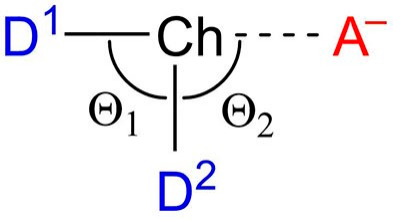
Chalcogen‐bonded D_2_Ch⋅⋅⋅A^−^ model complexes (Chv=vO, S, Se, Te; D, A = F, Cl, Br).

In this study, we have computationally analyzed a range of chalcogen‐bonded D_2_Ch⋅⋅⋅A^−^ complexes (Ch = O, S, Se, Te; D, A = F, Cl, Br; see Scheme 1), using relativistic density functional theory (DFT) at ZORA‐M06/QZ4P. One purpose of our work is to provide a set of consistent structural and energy data from which reliable trends can be inferred for a wide range of model systems. The primary objective is to achieve a detailed understanding of the nature of chalcogen bonds by studying the associated electronic structure and bonding mechanism and compare them with the better‐known halogen bonds and hydrogen bonds.^[9]^

To this end, we first explore how the geometries and energies of our model complexes D_2_Ch⋅⋅⋅A^−^ vary as the chalcogen atom (Ch), or the chalcogen bond accepting Lewis base (A^−^) are varied. To understand the origin of the computed trends, activation strain analyses^[10]^ are performed on the formation of the chalcogen‐bond complexes. As part of these analyses, the interaction energy and the underlying bonding mechanism are furthermore examined in the context of quantitative Kohn‐Sham molecular orbital (MO) theory in combination with an energy decomposition analysis (EDA).[^11^, ^12^] Our systematic and detailed analyses along the entire reaction profile for each of the chalcogen‐bond complexation reactions provide in‐depth insights. In particular, they demonstrate that chalcogen bonds are not at all purely electrostatic phenomena but are, to a substantial extent, covalent in nature.

## 2 Theoretical Methods

### 2.1 Computational Details

All calculations were carried out using the Amsterdam Density Functional (ADF) 2017.103 program.^[13]^ The equilibrium geometries and energies of chalcogen‐bonded complexes were computed at DFT level using the meta‐hybrid functional M06.^[14]^ In addition, a large uncontracted relativistically optimized QZ4P Slater type orbitals (STOs) basis set containing diffuse functions was used. The QZ4P all‐electron basis set,^[15]^ no frozen‐core approximation, is of quadruple‐*ζ* quality for all atoms and has been augmented with the following sets of polarization and diffuse functions: two 3 *d* and two 4 *f* on oxygen and fluorine, three 3 *d* and two 4 *f* on sulfur and chlorine, two 4 *d* and three 4 *f* on selenium and bromine, one 5 *d* and three 4 *f* on tellurium and iodine. The molecular density was fitted by the systematically improvable Zlm fitting scheme. The scalar relativistic effects were accounted for by using the zeroth‐order regular approximation (ZORA) Hamiltonian.^[16]^ It has been shown that these computational settings give accurate bond lengths and energies.^[17]^

### 2.2 Analysis of the Bonding Mechanism

Insight into the bonding mechanism is obtained through activation strain analyses of the various chalcogen bond formation reactions. These complexation reactions are computationally modeled by increasing the distance between A^−^ and the Ch atom of the D_2_Ch fragment, allowing the system to geometrically relax at each point. The D_2_Ch⋅⋅⋅A^−^ distance is increased from the actual bond length value in the chalcogen‐bonded complex (*r*_Ch⋅⋅⋅A_^−^) to a value of 4.300 Å. Thus, each analysis starts from an optimized D_2_Ch⋅⋅⋅A^−^ complex, which is then transformed to the D_2_Ch molecule and a halide at a relatively large distance.

These complexation reactions are analyzed using the activation strain model. The activation strain model of chemical reactivity^[10]^ is a fragment‐based approach to understand the energy profile of a chemical process in terms of the original reactants. Thus, the potential energy surface Δ*E*(ζ) is decomposed along the reaction coordinate ζ (or just at one point along ζ) into the strain energy Δ*E*_strain_(ζ), which is associated with the geometrical deformation of the individual reactants as the process takes place, plus the actual interaction energy Δ*E*_int_(ζ) between the deformed reactants [Eq. 1].

(1) $$\Delta E\left(\zeta \right)\;=\;\Delta E{}_{\mathrm{strain}}\left(\zeta \right)\;+\;\Delta E{}_{\mathrm{int}}\left(\zeta \right)$$

In the equilibrium geometry, that is, for ζ = ζ_eq_, this yields an expression for the bond energy Δ*E*(ζ_eq_) = Δ*E*_strain_(ζ_eq_) + Δ*E*_int_(ζ_eq_). The PyFrag program was used to facilitate the analyses along the reaction coordinate ζ of the bond formation processes.^[18]^ The interaction energy Δ*E*_int_(ζ) between the deformed reactants is further analyzed in the conceptual framework provided by the quantitative Kohn–Sham MO model.^[11]^ To this end, it is decomposed in three physically meaningful terms [Eq. (2)] using a quantitative energy decomposition scheme developed by Ziegler and Rauk.^[12]^

(2) $$\Delta E{}_{\mathrm{int}}\left(\zeta \right)\;=\;\Delta V{}_{\mathrm{elstat}}\left(\zeta \right)\;+\;\Delta E{}_{\mathrm{Pauli}}\left(\zeta \right)\;+\;\Delta E{}_{\mathrm{oi}}\left(\zeta \right)$$

(3) $$\Delta V{}_{\mathrm{elstat}}\left(\zeta \right)\;=\;\Delta V{}_{\mathrm{elstat},\rho 1\rho 2}\left(\zeta \right)\;+\;\Delta V{}_{\mathrm{elstat},n1\rho 2}\left(\zeta \right)\;+\Delta V{}_{\mathrm{elstat},\rho 1n2}\left(\zeta \right)\;+\;\Delta V{}_{\mathrm{elstat},n1n2}\left(\zeta \right)$$

The usually attractive term Δ*V*_elstat_ corresponds to the classical Coulomb interaction between the unperturbed charge distributions of the deformed reactants and has four components [Eq. (3)]: i) the electrostatic repulsion between the electron densities of fragments 1 and 2, Δ*V*_elstat,*ρ*1*ρ*2_; ii) the electrostatic attraction between the nucleus of fragment 1 and the electron density of fragment 2, Δ*V*_elstat,*n*1*ρ*2_; iii) the electrostatic attraction between the electron density of fragment 1 and the nucleus of fragment 2, Δ*V*_elstat,*ρ*1*n*2_; and iv) the electrostatic repulsion between the nuclei of fragments 1 and 2, Δ*V*_elstat,*n*1*n*2_. The Pauli repulsion energy (Δ*E*_Pauli_) comprises the destabilizing interactions between occupied orbitals of the reactants and is responsible for steric repulsion. The orbital‐interaction energy (Δ*E*_oi_) accounts for charge transfer, that is, the interaction between occupied orbitals of one fragment with unoccupied orbitals of the other fragment, including the interactions of the highest occupied and lowest unoccupied MOs (HOMO−LUMO), and polarization, that is, empty–occupied orbital mixing on one fragment, due to the presence of another fragment.

The electron density distribution is analyzed using the Voronoi deformation density (VDD) method for computing atomic charges.^[19]^ The VDD atomic charge on atom X in a molecule (*Q*_X_^VDD^) is computed as the (numerical) integral of the deformation density in the volume of the Voronoi cell of atom X [Eq. (4)]. The Voronoi cell of atom X is defined as the compartment of space bounded by the bond midplanes on and perpendicular to all bond axes between nucleus X and its neighboring nuclei.

(4) $$Q_{X}^{\mathrm{VDD}}=\;-\;\underset{\mathrm{Voronoi}\;\mathrm{cell}\;\mathrm{of}\;X}{\int^{\;}}\left[\rho \left(r\right)\;-\;\rho_{\mathrm{promolecule}}\left(r\right)\right]dr$$

Here, the deformation density is the difference between *ρ*(r), *i*. *e*., the electron density of the overall molecule or complex, and *ρ*_promolecule_(r) = Σ_Y_ *ρ*_Y_(r), *i*. *e*., the superposition of spherical average‐of‐configuration atomic densities *ρ*_Y_(r) of each atom Y in the fictitious promolecule without chemical interactions, in which all atoms are considered neutral. The interpretation of the VDD charge *Q*_Ch_^VDD^ is rather straightforward and transparent: instead of measuring the amount of charge associated with a particular atom Ch, *Q*_Ch_^VDD^ directly monitors how much charge flows out of (*Q*_Ch_^VDD^ > 0) or into (*Q*_Ch_^VDD^ < 0) the Voronoi cell of atom Ch due to chemical interactions.

The VDD scheme can also be used to directly compute how much charge flows into or out of an atomic Voronoi cell X in an overall complex (*e*. *g*., [D_2_Ch⋅⋅⋅A]^−^) relative to two (poly)atomic molecular fragments (*e*. *g*., D_2_Ch and A^−^), instead of spherical atoms, as shown in [Eq. 5].

(5) $${\Delta Q}_{X}^{\mathrm{VDD}}=\;-\;\underset{\mathrm{Voronoi}\;\mathrm{cell}\;\mathrm{of}\;X\;\mathrm{in}\;\mathrm{complex}}{\int^{\;}}\left[\rho_{\mathrm{complex}}\left(r\right)\;-\;\rho_{\mathrm{fragment}\;1}\left(r\right)-\;\rho_{\mathrm{fragment}\;2}\left(r\right)\right]dr$$

Δ*Q*_X_^VDD^ is a measure of how the atomic charge of atom X changes due to the bonding between the fragment. In this work, [Eq. (5)] is used to compute the flow of electrons from the halide A^−^ to the chalcogen‐bond donating molecule D_2_Ch (see Δ*Q*_D2Ch_^VDD^ in Table 1).

**Table 1:** Activation strain analyses (in kcal mol^−1^) of a representative set of D_2_Ch⋅⋅⋅A^−^ at the equilibrium geometries (in Å, deg.)^[a]^

D_2_Ch⋅⋅⋅A^−^	Δ*E*	Δ*E*_strain_	Δ*E*_int_	Δ*Q*_D2Ch_^VDD^	*r*_Ch⋅⋅⋅A_	Δ*r*_D1−Ch_	Δ*r*_D2−Ch_	Θ_1_	Θ_2_	ΔΘ_1_
F_2_O⋅⋅⋅F^−^	−21.9	28.3	−50.2	−0.37	1.784	0.408	−0.002	97.6	97.6	−6.1
F_2_O⋅⋅⋅Cl^−^	−9.9	28.1	−37.9	−0.35	2.183	0.402	0.007	98.8	101.3	−4.9
F_2_O⋅⋅⋅Br^−^	−11.5	45.0	−56.5	−0.48	2.113	0.582	0.023	98.6	103.4	−5.1
										
Cl_2_O⋅⋅⋅F^−^	−16.0	24.3	−40.3	−0.41	1.838	0.500	−0.032	104.4	99.5	−7.9
Cl_2_O⋅⋅⋅Cl^−^	−6.5	24.5	−31.0	−0.41	2.172	0.491	−0.012	105.5	105.5	−6.6
Cl_2_O⋅⋅⋅Br^−^	−11.0	46.5	−57.5	−0.62	1.966	0.878	0.009	105.7	110.8	−6.6
										
Br_2_O⋅⋅⋅F^−^	−12.9	4.5	−17.4	−0.26	2.162	0.153	0.007	106.1	86.6	−8.0
Br_2_O⋅⋅⋅Cl^−^	−6.0	1.5	−7.6	−0.19	2.673	0.084	0.021	111.9	94.0	−2.2
Br_2_O⋅⋅⋅Br^−^	−6.2	20.6	−26.7	−0.44	2.243	0.425	0.026	108.2	108.2	−5.9
										
F_2_S⋅⋅⋅F^−^	−50.1	16.2	−66.3	−0.35	1.813	0.227	0.045	87.0	87.0	−11.2
F_2_S⋅⋅⋅Cl^−^	−23.1	8.5	−31.7	−0.21	2.452	0.149	0.031	89.3	88.2	−8.9
F_2_S⋅⋅⋅Br^−^	−19.8	7.1	−26.9	−0.19	2.647	0.135	0.028	90.1	88.7	−8.1
										
Cl_2_S⋅⋅⋅F^−^	−53.1	24.5	−77.5	−0.47	1.748	0.461	0.035	92.1	93.0	−11.5
Cl_2_S⋅⋅⋅Cl^−^	−25.3	14.8	−40.1	−0.34	2.339	0.323	0.032	94.4	94.4	−9.2
Cl_2_S⋅⋅⋅Br^−^	−22.4	14.4	−36.9	−0.34	2.506	0.322	0.030	94.9	95.1	−8.7
										
Br_2_S⋅⋅⋅F^−^	−51.7	22.2	−73.9	−0.51	1.743	0.487	0.028	92.8	93.9	−11.1
Br_2_S⋅⋅⋅Cl^−^	−24.7	12.2	−36.8	−0.36	2.346	0.320	0.027	95.6	95.3	−8.3
Br_2_S⋅⋅⋅Br^−^	−22.3	12.3	−34.6	−0.36	2.507	0.327	0.027	96.0	96.0	−7.9
										
F_2_Te⋅⋅⋅F^−^	−72.4	7.9	−80.3	−0.32	2.054	0.162	0.038	84.2	84.2	−9.7
F_2_Te⋅⋅⋅Cl^−^	−42.5	5.9	−48.5	−0.24	2.608	0.134	0.035	85.2	86.3	−8.7
F_2_Te⋅⋅⋅Br^−^	−38.1	5.6	−43.7	−0.24	2.777	0.130	0.034	85.2	86.6	−8.7
										
Cl_2_Te⋅⋅⋅F^−^	−73.3	10.8	−84.1	−0.39	2.039	0.285	0.047	90.0	88.5	−8.2
Cl_2_Te⋅⋅⋅Cl^−^	−43.0	8.6	−51.6	−0.30	2.582	0.249	0.045	91.4	91.4	−6.8
Cl_2_Te⋅⋅⋅Br^−^	−38.6	8.4	−47.1	−0.30	2.745	0.246	0.045	91.4	91.8	−6.8
										
Br_2_Te⋅⋅⋅F^−^	−72.0	9.9	−81.9	−0.41	2.040	0.227	0.045	90.8	88.5	−8.3
Br_2_Te⋅⋅⋅Cl^−^	−42.0	7.6	−49.6	−0.32	2.582	0.461	0.035	92.5	92.0	−6.6
Br_2_Te⋅⋅⋅Br^−^	−37.7	7.6	−45.3	−0.32	2.751	0.487	0.028	92.8	92.8	−6.3

[a] Computed at ZORA‐M06/QZ4P. For full set of data, see Tables S1–S2 in the Supporting Information.

## 3 Results and Discussion

### 3.1 Chalcogen Bond Strength and Structure

The results of our ZORA‐M06/QZ4P calculations are shown in Table 1 for a representative selection of oxygen‐, sulfur‐, and tellurium‐bonded model complexes D_2_Ch⋅⋅⋅A^−^, covering D, A = F, Cl, and Br (the complete dataset for all model systems is provided in Tables S1–S2). In the first place, we note that all model reactions are associated with single‐well potential energy surfaces (PES), that is, there is no energy barrier separating the reactants from their resulting complex. In the cases where D ≠ A, *C*_s_ symmetric complexes with D^1^−Ch bond lengths longer than D^2^−Ch and with bond angles Θ_1_ ≠ Θ_2_ are formed. For the cases where D = A, *C*_2v_ symmetric complexes with equal bond distances *r*_Ch−D1_ = *r*_Ch⋅⋅⋅A_ and equal bond angles Θ_1_ = Θ_2_ are formed (see Table 1).

In general, chalcogen bonds become stronger on descending group 16 in the periodic table, in agreement with previous *ab initio* results.[^4b^, ^17^] The heavier D_2_Ch⋅⋅⋅A^−^ chalcogen bonds (*i*. *e*., Ch = S, Se, and Te) become weaker and longer as the accepting halide (A^−^) varies from F^−^ to Br^−^. In the case of the tellurium‐bonded complexes D_2_Te⋅⋅⋅A^−^, for example, the Δ*E* weakens from around −73 kcal mol^−1^ for A^−^ = F^−^ to around −38 kcal mol^−1^ for A^−^ = Br^−^ (see Table 1). However, the oxygen chalcogen bonds D_2_O⋅⋅⋅A^−^ display a more complex dependency of Δ*E* upon variation of the accepting halide A^−^. From A^−^ = F^−^ to Cl^−^, the oxygen‐bond strength still weakens, similar to the situation for the heavier chalcogen bonds. However, thereafter, from A^−^ = Cl^−^ to Br^−^, the oxygen‐bond strength does not weaken but instead becomes stronger. This is most clearly seen in the series constituted by the complex F_2_O⋅⋅⋅A^−^ between an oxygen molecule and a halide ion. Here, Δ*E* for the oxygen bond strength varies along A^−^ = F^−^, Cl^−^, and Br^−^ with values of −21.9, −9.9, and −11.5 kcal mol^−1^, respectively (see Table 1).

When the substituent D is varied from D = F to D = Br, the heavier chalcogen‐bond strength (*i*. *e*. Ch = S, Se, and Te) changes only slightly (see Table 1 and S1 in the Supporting Information). For example, along the series from F_2_Te⋅⋅⋅F^−^ to Br_2_Te⋅⋅⋅F^−^, the tellurium bond strength varies only from a Δ*E* value of −72.4 to −72.0 kcal mol^−1^, the tellurium‐bond distance *r*_Ch⋅⋅⋅A_ decreases in value from 2.054 to 2.040 Å, and the stretch Δ*r*_D1−Ch_ increases in value from 0.162 to 0.227 Å. An exception to this is again the oxygen bond D_2_O⋅⋅⋅A^−^, which becomes weaker and longer as D is varied from F to Br (see Table 1). For example, along the series from F_2_O⋅⋅⋅F^−^ to Br_2_O⋅⋅⋅F^−^, the oxygen‐bond strength weakens from a Δ*E* value of −21.9 to only −12.9 kcal mol^−1^, the oxygen‐bond distance *r*_Ch⋅⋅⋅A_ increases in value from 1.784 to 2.162 Å, and the stretch Δ*r*_D1−Ch_ decreases in value from 0.408 to 0.153 Å.

### 3.2 Bond Analyses with Variation of Ch

**Figure 1:**
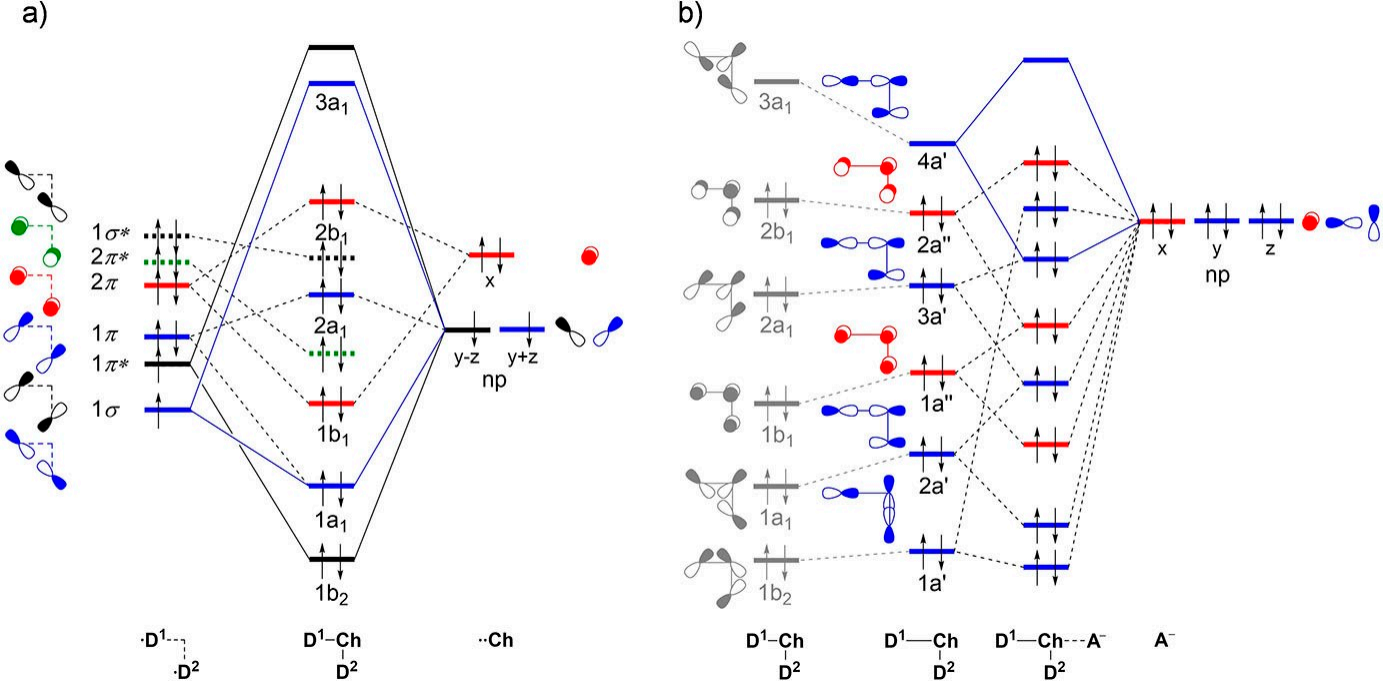
Schematic molecular orbital diagram for a) isolated D_2_Ch fragments at *C*_2v_ symmetry (blue: a_1_; green: a_2_; red: b_1_; black: b_2_) and b) D_2_Ch⋅⋅⋅A^−^ complexes. The first column in (b) refers to the isolated D_2_Ch fragment and the second column refers to the D_2_Ch fragment deformed to its *C*_s_ symmetric geometry in the complex (blue: a′; red: a′′), in which one D−Ch bond has been elongated. See Figure S1 in the Supporting Information for the 3D isosurfaces of the orbitals.

The strengthening of chalcogen bonds D_2_Ch⋅⋅⋅A^−^, as Ch varies along O, S, Se, and Te, with no change in the donating atom (D) and the accepting halide (A^−^), is related to the increasing electronegativity difference across the D−Ch bonds as Ch descends in the periodic table, which is translated into two main effects. Firstly, this causes the Ch atom to become increasingly positive along O, S, Se, and Te (see VDD atomic charges in Table 2), resulting in a greater electrostatic attraction. Secondly, this causes, among other effects that will be explained later, the σ* D−Ch antibonding 4a′ acceptor orbital to have higher amplitude on Ch (see Figure 1), resulting in stronger HOMO−LUMO orbital interactions.

**Table 2:** Bond lengths (in Å), bond angle (in deg.), VDD charge (in a.u.), orbital energies (in eV) and the homolytic bond dissociation energy without ZPE (in kcal mol^−1^) of isolated D_2_Ch fragments.^[a]^

D_2_Ch	*r*_D−Ch_	Θ_1_	*Q*_Ch_^VDD^	ϵ(1a_1_)	ϵ(3a_1_)	ϵ(1b_1_)	ϵ(2b_1_)	BDE_D−Ch_^[b]^
F_2_O	1.376	103.7	0.09	−16.6	−2.0	−15.8	−9.6	38.0
Cl_2_O	1.681	112.3	−0.06	−13.8	−3.3	−13.2	−8.1	34.9
Br_2_O	1.818	114.1	−0.13	−12.6	−3.3	−12.0	−7.7	33.7
								
F_2_S	1.586	98.2	0.18	−15.0	−0.7	−14.5	−7.1	92.2
Cl_2_S	2.016	103.6	0.12	−12.1	−2.5	−11.4	−7.1	64.0
Br_2_S	2.180	103.9	0.04	−11.3	−2.7	−10.5	−7.0	51.4
								
F_2_Se	1.730	96.3	0.28	−13.9	−1.6	−13.4	−7.2	87.6
Cl_2_Se	2.155	101.1	0.21	−11.6	−2.7	−10.8	−7.0	63.2
Br_2_Se	2.312	101.7	0.13	−10.8	−2.8	−10.1	−6.9	50.6
								
F_2_Te	1.892	93.9	0.31	−13.0	−2.0	−12.7	−6.6	92.8
Cl_2_Te	2.333	98.2	0.29	−11.0	−2.6	−10.3	−6.6	69.3
Br_2_Te	2.492	99.1	0.22	−10.3	−2.7	−9.6	−6.4	55.7

[a] Computed at ZORA‐M06/QZ4P; [b] Energy for the reaction D_2_Ch→DCh^.^+D^.^.

The trend in bond strength Δ*E* is mainly determined by the interaction energy Δ*E*_int_. For example, from F_2_O⋅⋅⋅F^−^ to F_2_Te⋅⋅⋅F^−^, Δ*E* is strengthened from −21.9 to −72.4 kcal mol^−1^ while Δ*E*_int_ is strengthened from −50.2 to −80.3 kcal mol^−1^ (see Table 1). The trend in Δ*E* is further enhanced by the strain energy (Δ*E*_strain_), which becomes less destabilizing from Ch = O to Te. However, the differences are smaller than the differences in Δ*E*_int_. For example, from F_2_O⋅⋅⋅F^−^ to F_2_Te⋅⋅⋅F^−^, Δ*E*_strain_ is weakened by 20.4 kcal mol^−1^ (from 28.3 to 7.9 kcal mol^−1^; see Table 1), while Δ*E*_int_ becomes 30.1 kcal mol^−1^ more stable.

**Figure 2:**
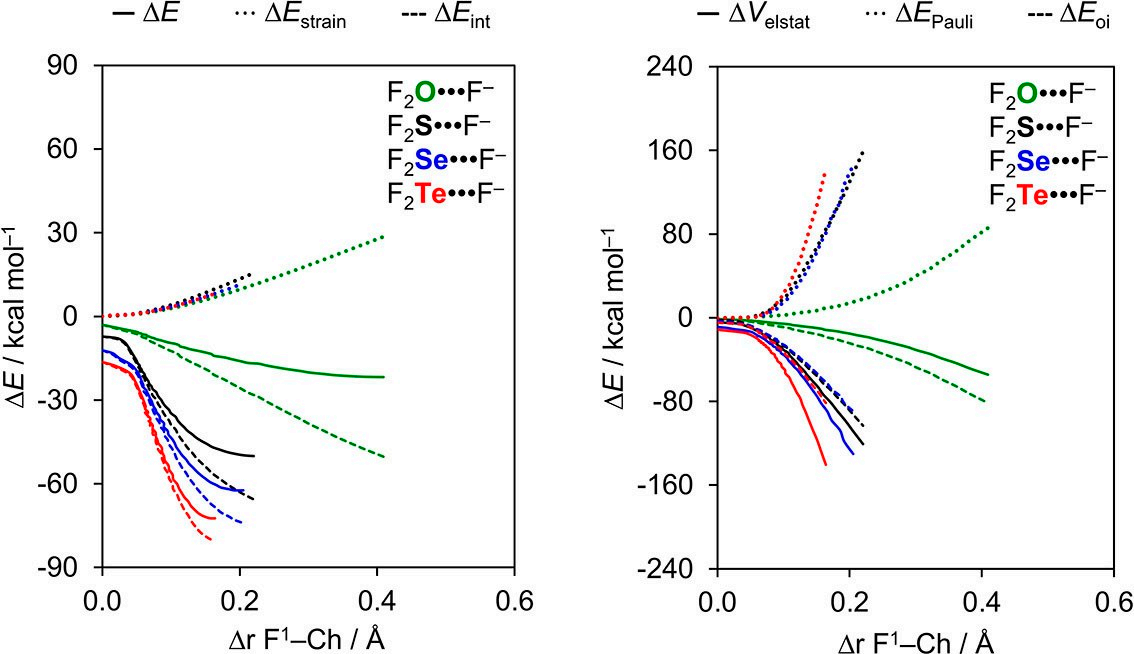
Activation strain (left panel) and energy decomposition (right panel) analyses of F_2_Ch⋅⋅⋅F^−^ chalcogen‐bonded complexes (green, Ch = O; black, Ch = S; blue, Ch = Se; red, Ch = Te).

To understand the origin of these trends, we have carried out activation strain analyses along the entire reaction coordinate ζ, projected onto the stretch in D^1^−Ch bond, Δ*r*_Ch−D1_, that occurs as the chalcogen‐bond accepting A^−^ atom approaches the D_2_Ch molecule (see Theoretical Methods section). The resulting activation strain diagrams (ASD) including EDA terms of the interaction are shown for a representative example series, namely, F_2_O⋅⋅⋅F^−^ to F_2_Te⋅⋅⋅F^−^, in Figure 2 (for the complete dataset, see Table S2). Again, the trend in bond energy Δ*E* is mainly determined by Δ*E*_int_(ζ), which strengthens when going from Ch = O to Te (Figure 2, left). On the other hand, the Δ*E*_strain_(ζ) curves almost coincide. However, the strain curves reach a final point at ζ_eq_, that is, the equilibrium geometry of the complex; and here the strain energy Δ*E*_strain_(ζ_eq_) becomes more destabilizing from Ch = Te to O. Note that the trend in strain energies at the equilibrium geometries along the series of F_2_Ch⋅⋅⋅F^−^ complexes (see Table 1) arises mainly from changes in the steepness of the interaction curves, not from the relatively minor variation in the strain curves (see Figure 2). Thus, as the F_2_Ch⋅⋅⋅F^−^ interaction gets weaker along Ch = Te, Se, S and O, the interaction curve becomes shallower and the balance between strain and interaction curve, *i*. *e*., the stationary point of the complex, occurs at longer and longer F−Ch distances and, consequently, more destabilizing Δ*E*_strain_(ζ_eq_) (see Table 1).

**Figure 3:**
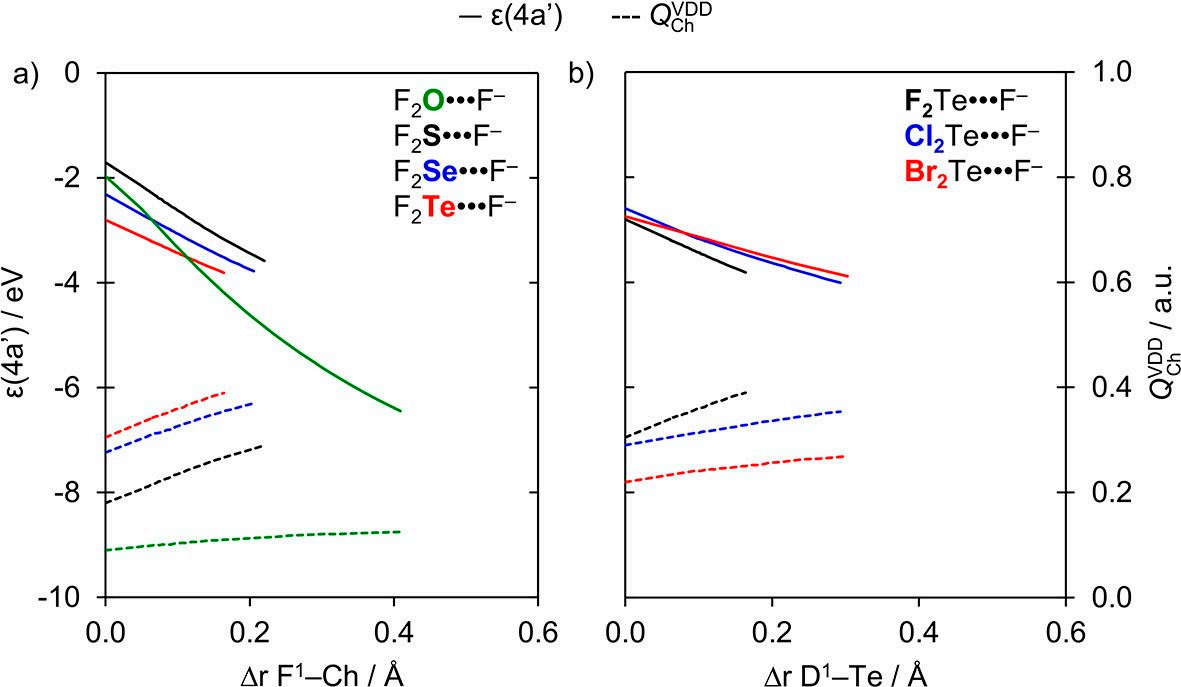
Energy of the 4a′ orbital (in eV) and the VDD charge on Ch atom (in a.u.) in the neutral fragment D_2_Ch projected onto a) the F^1^−Ch bond stretch (green, Ch = O; black, Ch = S; blue, Ch = Se; red, Ch = Te) and b) the D^1^−Te bond stretch (black, D = F; blue, D = Cl; red, D = Br).

**Figure 4:**
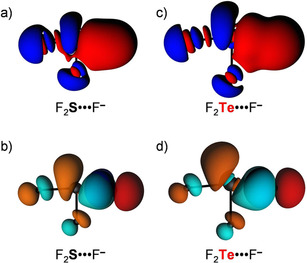
Deformation density (Δ*ρ*(r)=*ρ*_[D2Ch⋅⋅⋅A_‐_]_(r)−*ρ*_D2Ch_(r)−*ρ*_A_‐(r); red = depletion; blue = accumulation) plot (a and c) and HOMO−LUMO interaction (b and d) for a representative series of D_2_Ch⋅⋅⋅A^−^ chalcogen bonds.

To understand the trends in Δ*E*_int_(ζ), we further decomposed the Δ*E*_int_ into the individual energy components (Figure 2, right). The electrostatic energy Δ*V*_elstat_(ζ) is the least stabilizing for Ch = O and then strengthens along S, Se, and Te. This can be understood by the increasing differences in electronegativity across the D−Ch bonds when going from O to Te, resulting in a larger positive charge on Ch. For example, the VDD atomic charge on Ch in F_2_O, F_2_S, F_2_Se, and F_2_Te amounts to +0.09, +0.18, +0.28, and +0.31 a.u., respectively, and becomes even more positive as the D^1^−Ch bond elongates (see Figure 3a). Nevertheless, our analyses reveal that the chalcogen bonding mechanism is absolutely not purely electrostatic but instead has a relatively large covalent component (Δ*E*_oi_), stemming mainly from the HOMO−LUMO interaction between the occupied halide np_y_ atomic orbital (AO) and the σ* D−Ch antibonding 4a′ acceptor orbital (see Figure 1). The associated charge transfer from A^−^ to D_2_Ch is reflected by the Δ*Q*_D2Ch_^VDD^, which is negative, *i*. *e*., D_2_Ch gains charge from A^−^ upon complexation, for all D_2_Ch⋅⋅⋅A^−^ complexes (see Table 1). For example, Δ*Q*_D2Ch_^VDD^ is −0.37 a.u. for F_2_O⋅⋅⋅F^−^ and −0.32 a.u. for F_2_Te⋅⋅⋅F^−^. The HOMO−LUMO charge transfer nature of the chalcogen bond is also clearly reflected by the associated deformation density. This is illustrated by the 3D plots of the deformation densities associated with chalcogen‐bond formation in F_2_S⋅⋅⋅F^−^ and F_2_Te⋅⋅⋅F^−^ (see Figure 4). As can be seen, there is charge depletion on the Lewis base F^−^ (and in between the Ch⋅⋅⋅F^−^ bond due to the Pauli repulsion^[10a]^) and charge accumulation on D_2_Ch. Note the 3D shape of the regions of charge depletion and accumulation: they reflect the shape of the 2p‐type lone pair from which the F^−^ Lewis base donates and the σ* D−Ch antibonding 4a′ acceptor orbital on D_2_Ch into which this charge is donated, respectively, in the HOMO−LUMO interaction. For the chalcogen bonded complexes, the orbital interaction term ranges from 37 % for F_2_Te⋅⋅⋅F^−^ to as much as 76 % for Br_2_O⋅⋅⋅F^−^ of the total bonding interactions (Δ*E*_oi_ + Δ*V*_elstat_; see Table S2 in the Supporting Information). As can be seen in our energy decomposition diagram, the orbital interaction curves Δ*E*_oi_(ζ) become more stabilizing from Ch = O to Te (Figure 2, right). The stronger orbital interaction for the heavier chalcogens is the result of the larger LUMO−HOMO overlap (*i*. *e*. ⟨4a′|np_y_⟩; see Figure 1 for the MO diagram which shows the np_y_ orbital of A^−^ pointing towards the D^1^−Ch bond of the D_2_Ch fragment) as Ch becomes more electropositive. For example, in the Cl_2_Ch⋅⋅⋅Cl^−^ series, ⟨4a′|np_y_⟩ increases from 0.12 to 0.20 to 0.22 to 0.24 along Ch = O, S, Se, and Te in the equilibrium geometry (see Table S2 in the Supporting Information). The larger percent contribution of the covalent component on oxygen bonds is simply because the electrostatic attraction is relatively weak, caused by the smaller positive charge on O (see Table 2).

Whereas Δ*E*_oi_(ζ) becomes more stabilizing from Ch = O to Te, it becomes comparable in magnitude for all chalcogens in the equilibrium geometry Δ*E*_oi_(ζ_eq_). This is a consequence of the fact that the F^1^−Ch bond expansion becomes more pronounced when going from Ch = Te to O. The increasing F^1^−Ch bond expansion causes the σ* D−Ch antibonding 4a′ acceptor orbital (see Figure 1a) to drop further in energy for lighter chalcogens, resulting in a smaller HOMO−LUMO gap and hence more stabilizing donor‐acceptor interactions. This effect can be observed in Figure 3a, which shows the energies of the σ* F−Ch antibonding 4a′ acceptor orbitals along the reaction coordinate. For Ch = S, Se, and Te, the energy of the σ* F−Ch antibonding 4a′ acceptor orbital converges to an energy value of −3.8 eV as the chalcogen bond is formed. For Ch = O, on the other hand, the σ* F−O antibonding 4a′ acceptor orbital energy quickly drops to a value of −6.4 eV, because the overlap between the F and O AOs is more sensitive to the D−Ch distance than for the more diffuse AOs of heavier Ch (see Figure S2 in the supporting information). However, it is counteracted by the orbital overlap between the σ* D−O antibonding 4a′ acceptor orbital and the np_y_ donor orbital, which is significantly worse for Ch = O than for other chalcogen systems (see Table S2 in the Supporting Information).

### 3.3 Bond Analyses with Variation of A^−^

Our analyses show that the weakening of heavier chalcogen bonds D_2_Ch⋅⋅⋅A^−^ (Ch = S, Se, Te), as the accepting group varies from A^−^ = F^−^ to Br^−^, is directly related to the concomitant reduction in electron‐donating capacity of the np‐type HOMO and thus the Lewis basicity of the A^−^ halide.^[20]^ We recall the chalcogen bonds display both an electrostatic component (Δ*V*_elstat_) and a covalent component (Δ*E*_oi_). The latter stems mainly from the HOMO−LUMO interaction between the occupied halide np atomic orbital (AO) and the σ* D−Ch antibonding 4a′ acceptor orbital (see Figure 1). Both Δ*V*_elstat_ and Δ*E*_oi_ are weakened as the halide HOMO becomes more diffuse and effectively lower in energy from A^−^ = F^−^ to Br^−^ (see Table S2).^[20b]^ Consequently, the interaction energy (Δ*E*_int_) and, thus, the net chalcogen‐bond strength Δ*E* becomes less stabilizing along A^−^ = F^−^ to Br^−^ (see Table 1 and Table S1 in the Supporting Information). This is very similar to what was found for hydrogen bonds DH⋅⋅⋅A^−^ and heavier halogen bonds DX⋅⋅⋅A^−^ (X = Cl, Br, I).^[9]^

**Figure 5:**
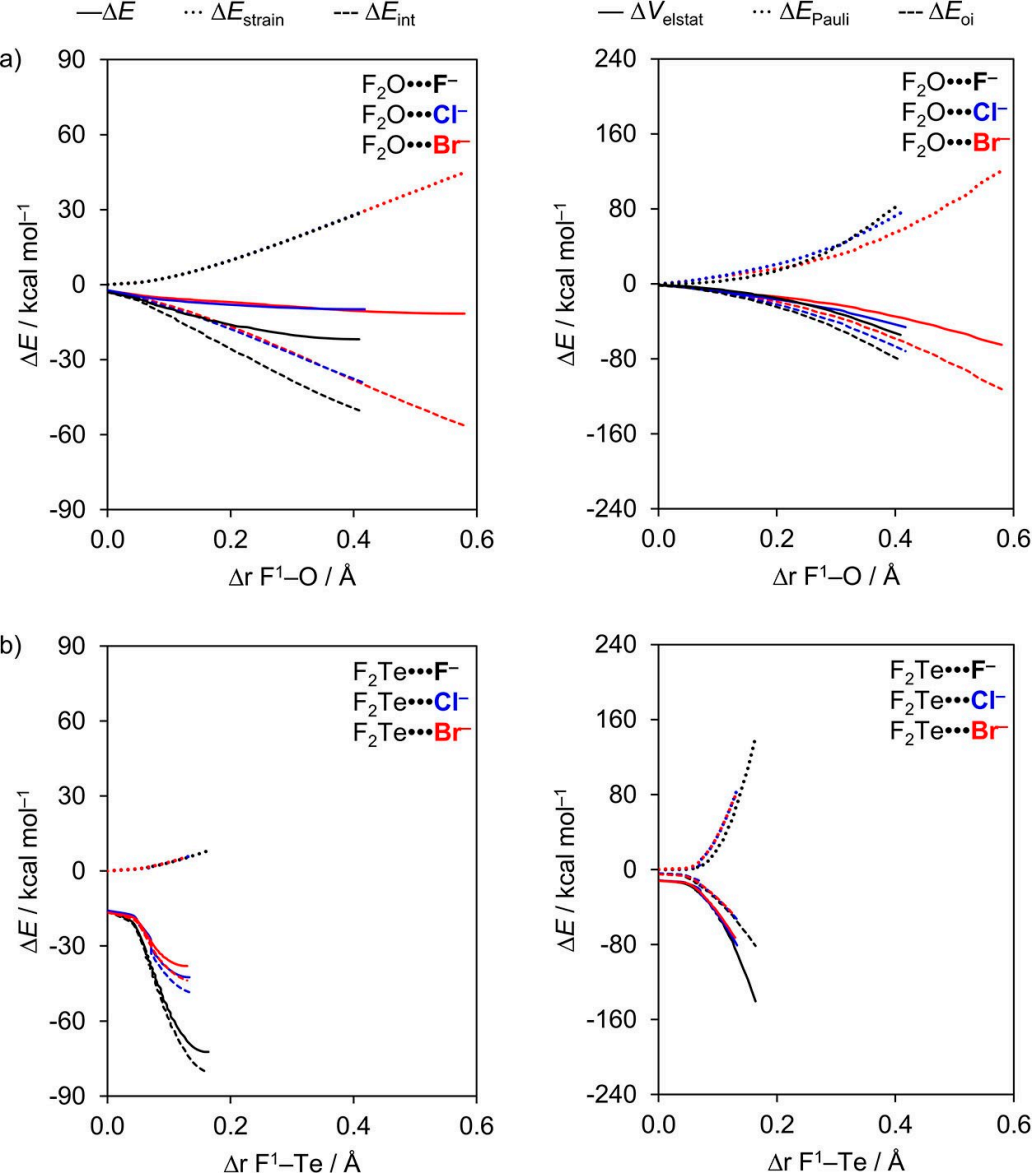
Activation strain (left panel) and energy decomposition (right panel) analyses of a) F_2_O⋅⋅⋅A^−^ and b) F_2_Te⋅⋅⋅A^−^ (black, A^−^ = F^−^; blue, A^−^ = Cl^−^; red, A^−^ = Br^−^).

The key to understanding why oxygen bonds D_2_O⋅⋅⋅A^−^ show a more complex, partially opposite trend (*i*. *e*., the expected weakening from A^−^ = F^−^ to Cl^−^ but thereafter a strengthening along A^−^ = Cl^−^ to Br^−^) is contained in the counteracting effects evolving from D−O bond stretching induced in the triatomic D_2_O molecule as it interacts with the halide A^−^. Interestingly, activation strain analyses reveal, again, that interaction energies recover the original trend in total energies, that is, Δ*E*_int_(ζ) weakens from A^−^ = F^−^ to Br^−^. This can be seen in Figure 5 which shows the activation strain and energy decomposition diagrams along the reaction coordinate ζ projected onto the stretch Δ*r*_D1−Ch_ for two representative series. Each diagram in Figure 5 refers to one particular F_2_O or F_2_Te molecule forming a chalcogen bonding with A^−^ = F^−^, Cl^−^, and Br^−^. The Δ*E*_strain_ curves within each subgraph coincide because they refer to the same D−Ch bond in the same triatomic molecule being stretched as the complexation reaction progresses. Consequently, the trend A^−^ = F^−^ to Br^−^ in the total F_2_O⋅⋅⋅A^−^ and F_2_Te⋅⋅⋅A^−^ energy profiles Δ*E* in each subgraph is directly determined by the trend in the corresponding Δ*E*_int_ curves.

The reason why the oxygen bonds D_2_O⋅⋅⋅A^−^ do not experience a weakening in Δ*E*_int_ from A^−^ = F^−^ to Br^−^, as all other chalcogen bonds, is promoted by a combination of factors: i) a weak D−O bond that is easily stretched; ii) a strong interaction with an approaching halide A^−^; and iii) a σ* D−Ch antibonding 4a′ acceptor orbital that drops in energy, more quickly than for other D^1^−Ch bonds due to a more sensitive overlap between the D^1^ and O AOs, as the D^1^−O bond elongates (see Figure 3 and Figure S2 in the supporting information). The latter generates a stronger driving force for D^1^−Ch stretching in D_2_Ch⋅⋅⋅A^−^ because this deformation enhances the orbital interactions and thus Δ*E*_int_. Note that, for D_2_O⋅⋅⋅A^−^, Δ*E*_oi_ is the strongest bonding component and that the Δ*E*_oi_(ζ) curves directly reflect the electron‐donating capacity of the np‐type HOMO of the A^−^ halides, that is, the Δ*E*_oi_ curves become more stabilizing from A^−^ = Br^−^ to F^−^ (see Figure 5). Indeed, D^1^−Ch stretching is most pronounced if this bond in the neutral fragment is weaker, that is, for the weaker chalcogen bonds (*e*. *g*., ca. 38 kcal mol^−1^ for F−O, ca. 35 kcal mol^−1^ for Cl−O and ca. 34 kcal mol^−1^ for Br−O; see Table 2). In this case, it is able to affect the trend in overall bond strength Δ*E*. The D^1^−O stretching in oxygen‐bonded complexes is most pronounced in the Cl_2_O⋅⋅⋅A^−^ series, along which the Cl^1^−O stretch Δ*r*_D1−Ch_ varies between 0.5 and 0.9 Å, but it is already relevant in the F_2_O⋅⋅⋅A^−^ series in which the F^1^−O stretch Δ*r*_Ch−D1_ varies between 0.4 and 0.6 Å from A^−^ = F^−^ to Br^−^ (see Table 1).

We conclude that, in general, chalcogen bonds D_2_Ch⋅⋅⋅A^−^ become weaker along A^−^ = F^−^ to Br^−^ because the larger radii and lower np AO energies of the halides lead to weaker electrostatic attraction and weaker orbital interactions. The trend in D_2_O⋅⋅⋅A^−^ oxygen bond strength is partially inverted, that is, Δ*E* becomes more stabilizing along A^−^ = Cl^−^ and Br^−^ because of a subtler interplay of factors. Notably, a significant stretching of the relatively weak D−O bonds in the D_2_O⋅⋅⋅A^−^ equilibrium structures lowers the σ* D−O antibonding 4a′ acceptor orbital and thus amplifies the donor‐acceptor orbital interactions.

### 3.4 Bond Analyses with Variation of D

**Figure 6:**
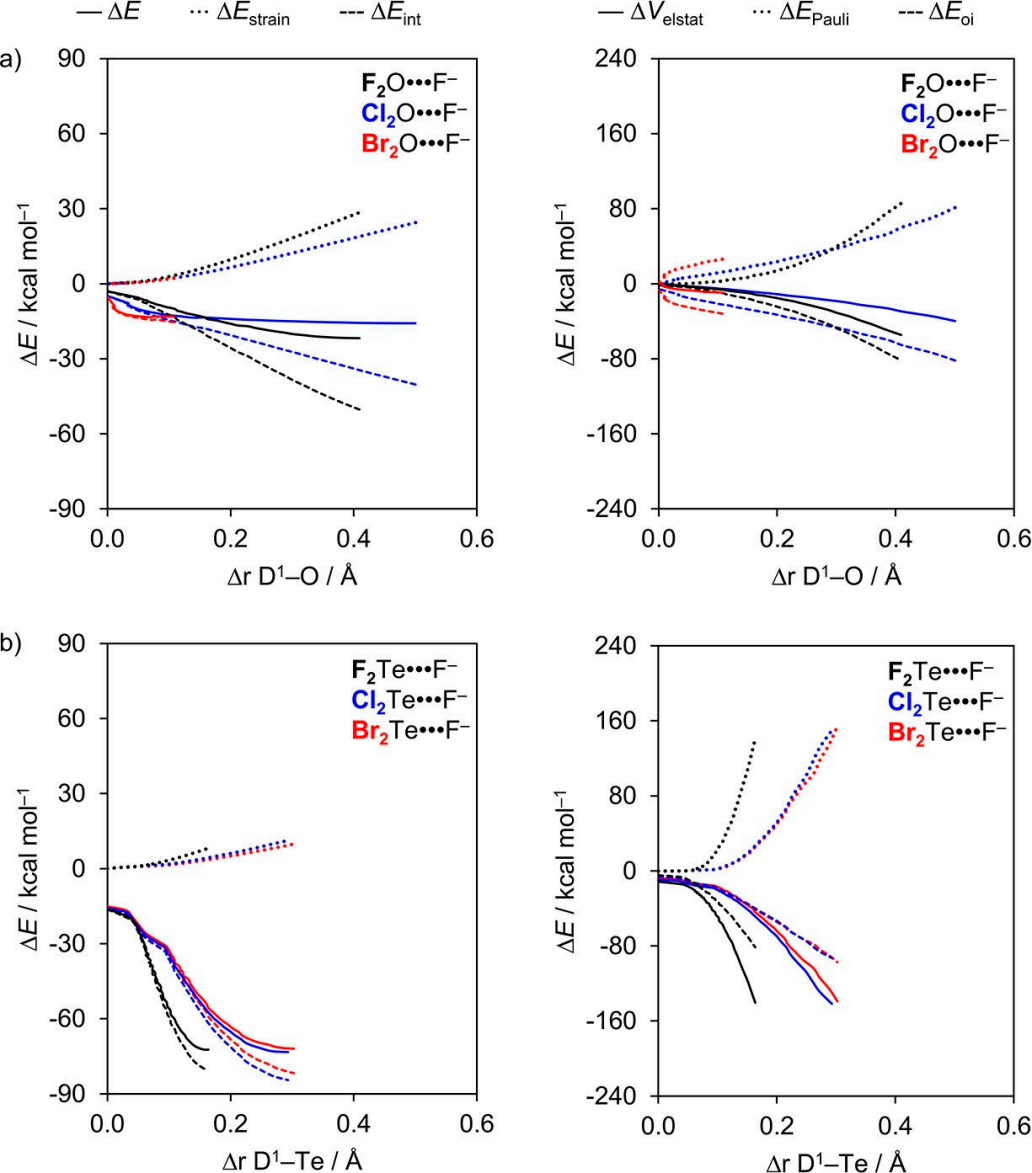
Activation strain (left panel) and energy decomposition (right panel) analyses of a) D_2_O⋅⋅⋅F^−^ and b) D_2_Te⋅⋅⋅F^−^ (black, D = F; blue, D = Cl; red, D = Br).

The strength of the heavier chalcogen bonds D_2_Ch⋅⋅⋅A^−^ varies little when going from D = F to Br because the Cl−Ch and Br−Ch bonds are significantly weaker than the F−Ch. This allows the Cl−Ch and Br−Ch bonds to stretch to a higher extent and, therefore, to have more stabilizing electrostatic attraction and orbital interactions. For the oxygen bonds D_2_O⋅⋅⋅A^−^, the bond energy is weakened along the same variation because the D−Ch bond strength are all comparable (see Table 2). In both cases, the trend in bond strength Δ*E* is determined by the interaction energy Δ*E*_int_. For example, from F_2_O⋅⋅⋅F^−^ to Br_2_O⋅⋅⋅F^−^, Δ*E*_int_ is weakened from −50.2 to −17.4 kcal mol^−1^, respectively, whereas from F_2_Te⋅⋅⋅F^−^ to Br_2_Te⋅⋅⋅F^−^, the bond energy only changes from −80.3 to −81.9 kcal mol^−1^, respectively (see Table 1). The strain energy (Δ*E*_strain_) is not negligible, but it does not offset the trend set by Δ*E*_int_. Our activation strain analyses explain the above differences between oxygen and heavier chalcogen bonds (see Figure 6).

Starting with some general observations, we find that for oxygen, as well as heavier chalcogen bonds, the Δ*E*_strain_ curves are most unfavorable when D = F and gradually become less destabilizing as the donating atom is varied along D = F, Cl, and Br (see Figure 6). Furthermore, for all D_2_Ch⋅⋅⋅A^−^ complexes, the Δ*E*_int_ curves become less stabilizing along D = F, Cl, and Br. The resulting energy profiles of D_2_Ch⋅⋅⋅A^−^ depend on the balance between both Δ*E*_strain_ and Δ*E*_int_, but the interaction energy curves already show a very similar trend to Δ*E*.

The slope and shape of the Δ*E*_strain_ curves is of course directly related to the D^1^−Ch bond strength of the neutral fragment, which in general becomes stronger as the polarity across the D−Ch bond increases^[21]^ (see Table 2). From F_2_Ch to Br_2_Ch, where Ch is S, Se or Te, the halogen‐chalcogen bond strength decreases significantly from a value of ca. 93 to 50 kcal mol^−1^ (Table 2). The corresponding halogen−oxygen bonds are all much weaker, and variations in the homolytic bond dissociation energy (BDE) are also much smaller. From F_2_O to Br_2_O, the bond strength decreases from 38.0 to 33.7 kcal mol^−1^. Thus, for the heavier chalcogen‐bonded complexes, where Ch is S, Se, or Te, the Δ*E*_strain_ curves show a pronounced reduction in slope from F_2_Ch to Br_2_Ch, which, in the corresponding chalcogen‐bonded complexes F_2_Ch⋅⋅⋅A^−^ to Br_2_Ch⋅⋅⋅A^−^, translates into an increasing stretch Δ*r*_D1−Ch_ of the neutral fragment. As the stretch Δ*r*_D1−Ch_ becomes larger from equilibrium structures F_2_Ch⋅⋅⋅A^−^ to Br_2_Ch⋅⋅⋅A^−^, the Δ*E*_int_ curves have been able to descend further, to lower, more stabilizing energies. This stabilization is, of course, related to the Δ*V*_elstat_ and Δ*E*_oi_. Note that the electrostatic attraction and orbital interaction curves become less stabilizing along D = F, Cl, and Br, but turn out to have comparable strength in the equilibrium structures, because the D^1^−Ch bonds have been increasingly stretched in the latter, that is, in Cl_2_Ch⋅⋅⋅A^−^ and Br_2_Ch⋅⋅⋅A^−^. The bonding components Δ*V*_elstat_ and Δ*E*_oi_ are the most stabilizing for D = F because of the larger difference in electronegativity across the D−Ch bonds (*vide supra*). However, the Δ*E*_oi_ is able to further stabilize for D = Cl and Br because, in the equilibrium structure of the chalcogen‐bonded complexes, the Cl−Ch and Br−Ch bonds expand to a higher extent, resulting in a stronger stabilization of their σ* D−Ch antibonding 4a′ acceptor orbitals (see Figure 3b). Furthermore, the VDD atomic charge on Ch becomes increasingly more positive as the D^1^−Ch bond expands, which translates into more stabilizing Δ*V*_elstat_ for D = Cl and Br in the equilibrium geometry. The final result is, thus, a comparable stability among heavier chalcogen bonds D_2_Ch⋅⋅⋅A^−^ complexes when the substituent D is varied from F to Br.

### 3.5 Chalcogen Bonds Versus Halogen and Hydrogen Bonds

**Figure 7:**
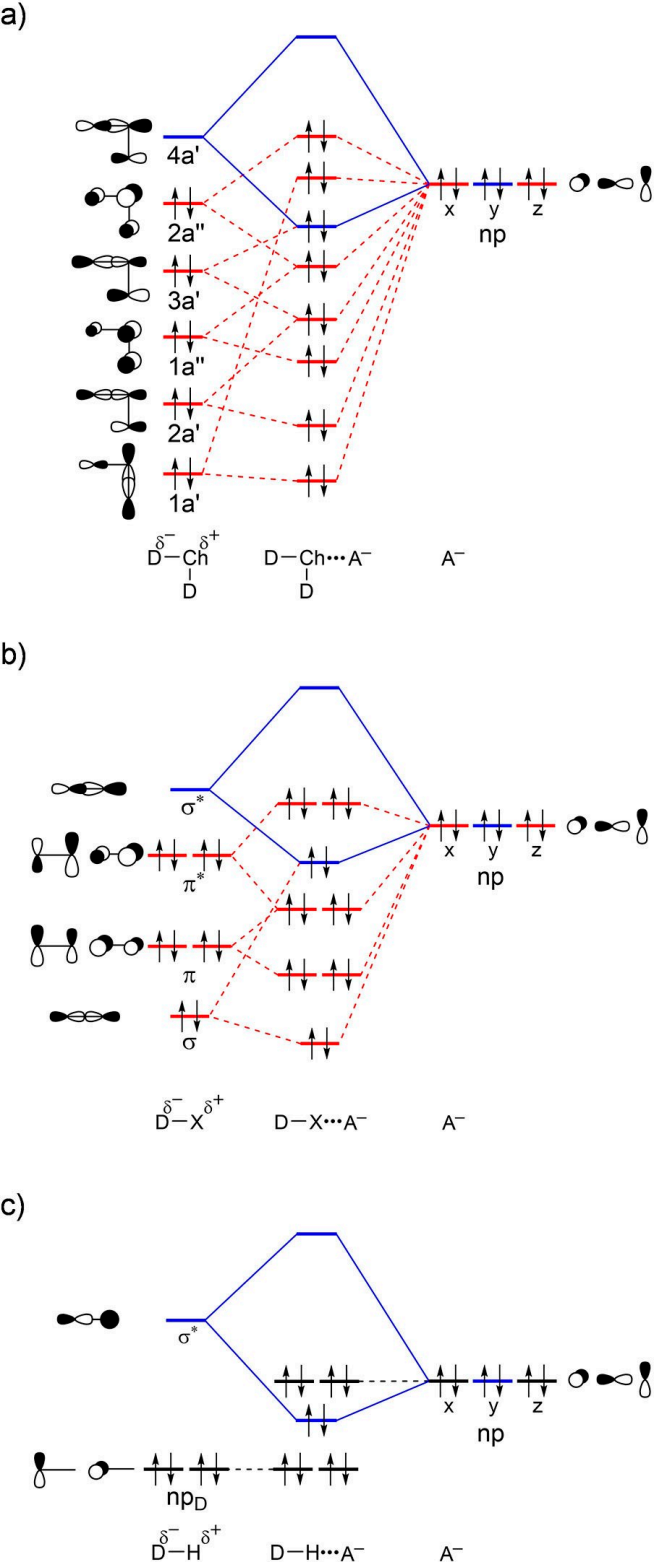
Generic molecular orbital diagrams for a) D_2_Ch⋅⋅⋅A^−^ chalcogen bonds, b) DX⋅⋅⋅A^−^ halogen bonds, and c) DH⋅⋅⋅A^−^ hydrogen bonds.

Our analyses highlight that chalcogen bonds, halogen bonds, and hydrogen bonds are all similar in nature.^[9]^ Each of these bonds in our set of model systems has a significant covalent component in addition to electrostatic attraction, and can range in strength roughly between −6 and −70 kcal mol^−1^ (see Figure 7). Chalcogen bonds and halogen bonds have a larger range in polarities in D−Ch and D−X than in D−H bonds and are in general stronger than hydrogen bonds because of more stabilizing orbital interactions (see Table S3 for bond energies Δ*E* of a representative series of XB and HB Δ*E* computed at ZORA‐M06/QZ4P). However, chalcogen bonds and halogen bonds also have more destabilizing Pauli repulsion because the lone‐pair HOMO of the Lewis base overlaps with more closed shells, in particular, with the σ D−Ch bonding 3a′ and 2a′ FMOs or σ D−X bonding FMO with a higher amplitude on Ch and X, respectively, than the amplitude of σ D−H bonding FMO has on H (see Figure 7; see also Ref. 9). Our analyses provide a unified picture for chalcogen bonds, halogen bonds, and hydrogen bonds based on quantitative Kohn‐Sham molecular orbital theory, which proves that these intermolecular interactions cannot be described by a pure and simple electrostatic model.

## 4 Conclusions

Chalcogen bonds in D_2_Ch⋅⋅⋅A^−^ range between 6 and 73 kcal mol^−1^ in strength, becoming stronger as the chalcogen atom becomes more electropositive, along Ch = O, S, Se and Te, and also as the halide becomes a stronger Lewis base, along A^−^ = Br^−^, Cl^−^ and F^−^. The trend upon variation of the substituent along D = F, Cl, Br is less pronounced, as are all trends for the relatively weak oxygen bonds. This follows from our bonding analyses based on relativistic density functional theory (DFT) calculations at ZORA‐M06/QZ4P.

Our activation strain and quantitative Kohn‐Sham MO bonding analyses reveal that the chalcogen bonds in D_2_Ch⋅⋅⋅A^−^ are similar in nature to halogen bonds in DX⋅⋅⋅A^−^ and hydrogen bonds in DH⋅⋅⋅A^−^ (Ch = O, S, Se, Te; D, X, A = F, Cl, Br). Chalcogen bonds are far from being solely electrostatic phenomena. Similar to halogen and hydrogen bonds, chalcogen bonds have a sizeable covalent component, ranging up to 80 % of the bonding components (Δ*V*_elstat_ + Δ*E*_oi_), stemming from HOMO−LUMO interactions between the np‐type lone pair on the bond accepting fragment A^−^ and the LUMO with strong D−Ch σ* anti‐bonding character on the bond donating fragment D_2_Ch.

Chalcogen bonds become stronger for heavier Ch because of the greater difference in electronegativity across the D−Ch bonds, causing: i) the σ* D−Ch antibonding 4a′ acceptor orbital to have higher amplitude on Ch, enhancing HOMO−LUMO orbital interactions; and ii) the Ch to become more positively charged, resulting in greater electrostatic attraction when descending in group 16 of the periodic table. The chalcogen bonds also become stronger for lighter A^−^ because the electron‐donating capacity of the np‐type HOMO (*i*. *e*. Lewis basicity) of the halides increases ascending group 17 in the periodic table. The trends for oxygen bonds, as well as along various D, are less pronounced because of counteracting effects or small values in bond strength.

## Conflict of interest

The authors declare no conflict of interest.

## Supporting information

As a service to our authors and readers, this journal provides supporting information supplied by the authors. Such materials are peer reviewed and may be re‐organized for online delivery, but are not copy‐edited or typeset. Technical support issues arising from supporting information (other than missing files) should be addressed to the authors.

Supplementary
