# Supplementary material for: A Quantitative Molecular Orbital Perspective of the Chalcogen Bond
Source: ChemistryOpen. 2021 Feb 17;10(4):391–401. doi: 10.1002/open.202000323 (PMC8015733; doi:10.1002/open.202000323)
Supplement: Supplementary file 1 — Supplementary [file OPEN-10-391-s001.pdf]

ChemistryOpen

Supporting Information

A Quantitative Molecular Orbital Perspective of the Chalcogen Bond

Lucas de Azevedo Santos, Stephanie C. C. van der Lubbe, Trevor A. Hamlin, Teodorico C. Ramalho,* and F. Matthias Bickelhaupt*

Table of Contents

Figure S1. Schematic FMO and DFT MO isosurfaces (at 0.04 a.u.) for the D_2Ch fragment in a) the ground state at C_{2v} symmetry and in b) the geometry of the complex at C_s symmetry, computed at ZORA-M06/QZ4P.

Figure S2. Orbital overlap between the $F^{1\cdot}$ and $F^2Ch^{\cdot}$ open shell fragments in the equilibrium geometries ($r_{D^1–Ch,\ eq}$) and with the F^1–Ch bond stretched by 0.16 Å ($r_{D^1–Ch,\ eq+0.16Å}$), computed at ZORA-M06/QZ4P.

Table S1. Activation strain analyses (in kcal mol^{-1}) of $D_2Ch{\bullet\bullet\bullet}A^-$ chalcogen bonds at the equilibrium geometries (in Å, deg.).

Table S2. Energy decomposition analyses (in kcal mol^{-1}) of $D_2Ch{\bullet\bullet\bullet}A^-$ chalcogen bonds at the equilibrium geometries.

Table S3. Activation strain and energy decomposition analyses (in kcal mol^{-1}) of $D_mZ{\bullet\bullet\bullet}A^-$ hydrogen bonds, halogen bonds, and chalcogen bonds at the equilibrium geometries.

Table S4. Cartesian coordinates and bonding energies (in kcal mol^{-1}) for all stationary points of $DO^{\cdot}$, D_2O and $D_2O{\bullet\bullet\bullet}A^-$ structures, computed at ZORA-M06/QZ4P.

Table S5. Cartesian coordinates and bonding energies (in kcal mol^{-1}) for all stationary points of $DS^{\cdot}$, D_2S and $D_2S{\bullet\bullet\bullet}A^-$ structures, computed at ZORA-M06/QZ4P.

Table S6. Cartesian coordinates and bonding energies (in kcal mol^{-1}) for all stationary points of $DSe^{\cdot}$, D_2Se and $D_2Se{\bullet\bullet\bullet}A^-$ structures, computed at ZORA-M06/QZ4P.

Table S7. Cartesian coordinates and bonding energies (in kcal mol^{-1}) for all stationary points of $DTe^{\cdot}$, D_2Te and $D_2Te{\bullet\bullet\bullet}A^-$ structures, computed at ZORA-M06/QZ4P.

Table S8. Cartesian coordinates and bonding energies (in kcal mol^{-1}) for all stationary points of FH, FX, $FH{\bullet\bullet\bullet}F^-$ and $FX{\bullet\bullet\bullet}F^-$ structures, computed at ZORA-M06/QZ4P.

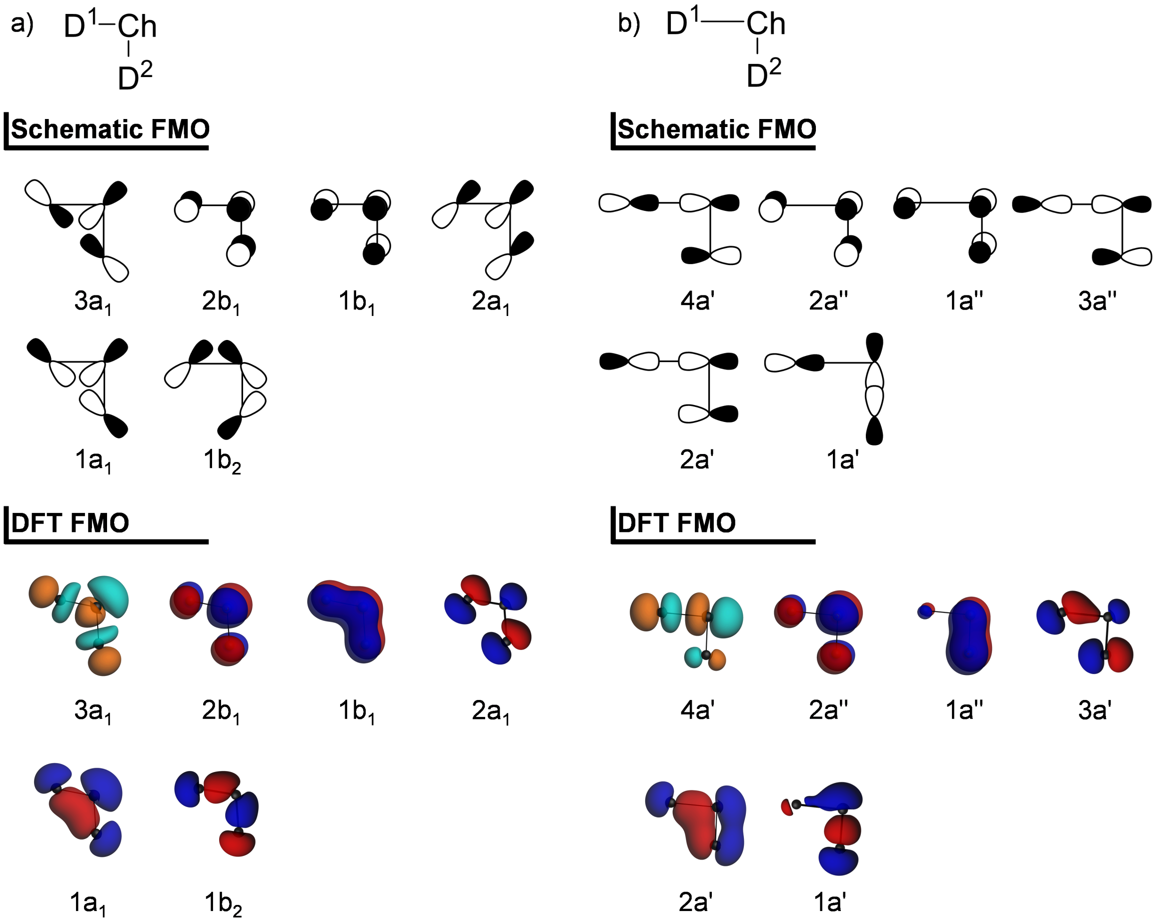

Figure S1. Schematic FMO and DFT MO isosurfaces (at 0.04 a.u.) for the D_2Ch fragment in a) the ground state at C_{2v} symmetry and in b) the geometry of the complex at C_s symmetry, computed at ZORA-M06/QZ4P.

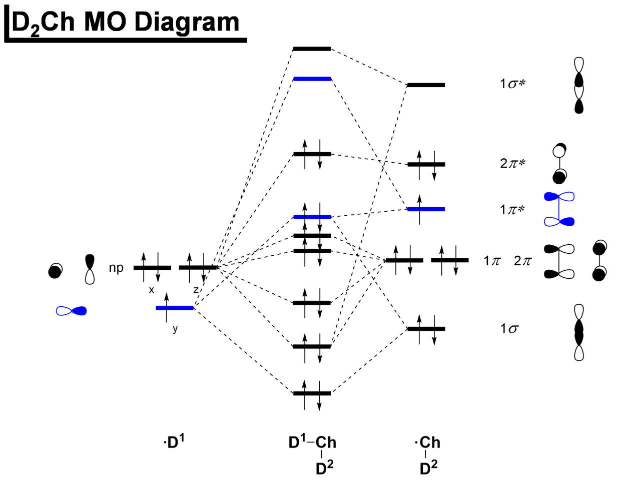

	⟨2p$_y$|1π*⟩	
	$r_{D^1\text{–Ch, eq}}$	$r_{D^1\text{–Ch, eq+0.16Å}}$
F_2O	0.19	0.17
F_2S	0.22	0.21
F_2Se	0.21	0.21
F_2Te	0.20	0.19

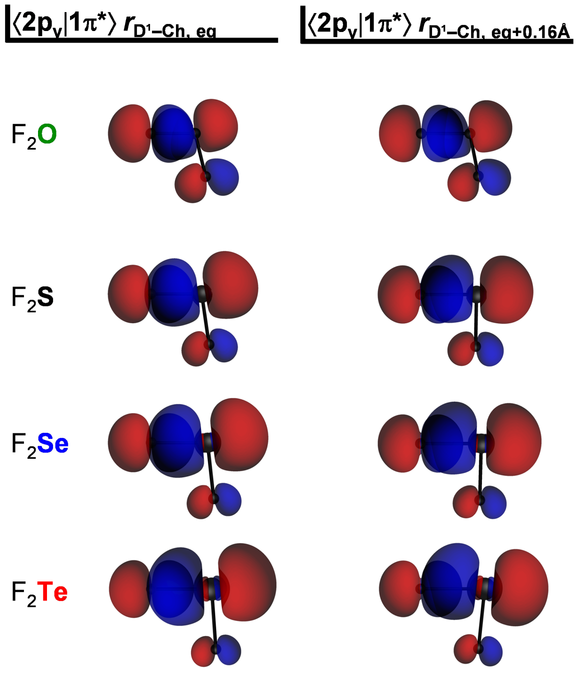

Figure S2. Orbital overlap between the F[1·] and F[2]Ch[·] open shell fragments in the equilibrium geometries ($r_{D^1\text{–Ch, eq}}$) and with the F[1]–Ch bond stretched by 0.16 Å ($r_{D^1\text{–Ch, eq+0.16Å}}$), computed at ZORA-M06/QZ4P.

Table S1. Activation strain analyses (in kcal mol^{-1}) of $D_2Ch{\cdots}A^-$ chalcogen bonds at the equilibrium geometries (in Å, deg.).[a]

$D_2Ch{\cdots}A^-$	$r_{Ch\cdots A}$	$r_{D^1–Ch}$	$r_{D^2–Ch}$	Θ_1	Θ_2	$\Delta r_{D^1–Ch}$	$\Delta r_{D^2–Ch}$	$\Delta\Theta_1$	ΔE	ΔE_{strain}	ΔE_{int}
$F_2O{\cdots}F^-$	1.784	1.784	1.374	97.6	97.6	0.408	–0.002	–6.1	–21.9	28.3	–50.2
$F_2O{\cdots}Cl^-$	2.183	1.778	1.383	98.8	101.3	0.402	0.007	–4.9	–9.9	28.1	–37.9
$F_2O{\cdots}Br^-$	2.113	1.958	1.399	98.6	103.4	0.582	0.023	–5.1	–11.5	45.0	–56.5
$Cl_2O{\cdots}F^-$	1.838	2.181	1.649	104.4	99.5	0.500	–0.032	–7.9	–16.0	24.3	–40.3
$Cl_2O{\cdots}Cl^-$	2.172	2.172	1.669	105.5	105.5	0.491	–0.012	–6.6	–6.5	24.5	–31.0
$Cl_2O{\cdots}Br^-$	1.966	2.559	1.690	105.7	110.8	0.878	0.009	–6.6	–11.0	46.5	–57.5
$Br_2O{\cdots}F^-$	2.162	1.971	1.825	106.1	86.6	0.153	0.007	–8.0	–12.9	4.5	–17.4
$Br_2O{\cdots}Cl^-$	2.673	1.902	1.839	111.9	94.0	0.084	0.021	–2.2	–6.0	1.5	–7.6
$Br_2O{\cdots}Br^-$	2.243	2.243	1.844	108.2	108.2	0.425	0.026	–5.9	–6.2	20.6	–26.7
$F_2S{\cdots}F^-$	1.813	1.813	1.631	87.0	87.0	0.227	0.045	–11.2	–50.1	16.2	–66.3
$F_2S{\cdots}Cl^-$	2.452	1.735	1.617	89.3	88.2	0.149	0.031	–8.9	–23.1	8.5	–31.7
$F_2S{\cdots}Br^-$	2.647	1.721	1.614	90.1	88.7	0.135	0.028	–8.1	–19.8	7.1	–26.9
$Cl_2S{\cdots}F^-$	1.748	2.477	2.051	92.1	93.0	0.461	0.035	–11.5	–53.1	24.5	–77.5
$Cl_2S{\cdots}Cl^-$	2.339	2.339	2.048	94.4	94.4	0.323	0.032	–9.2	–25.3	14.8	–40.1
$Cl_2S{\cdots}Br^-$	2.506	2.338	2.046	94.9	95.1	0.322	0.030	–8.7	–22.4	14.4	–36.9
$Br_2S{\cdots}F^-$	1.743	2.667	2.208	92.8	93.9	0.487	0.028	–11.1	–51.7	22.2	–73.9
$Br_2S{\cdots}Cl^-$	2.346	2.500	2.207	95.6	95.3	0.320	0.027	–8.3	–24.7	12.2	–36.8
$Br_2S{\cdots}Br^-$	2.507	2.507	2.207	96.0	96.0	0.327	0.027	–7.9	–22.3	12.3	–34.6
$F_2Se{\cdots}F^-$	1.931	1.931	1.773	87.3	87.3	0.201	0.043	–8.9	–62.4	11.4	–73.7
$F_2Se{\cdots}Cl^-$	2.495	1.888	1.765	88.6	89.6	0.158	0.035	–7.7	–34.6	7.7	–42.3
$F_2Se{\cdots}Br^-$	2.659	1.884	1.764	88.7	89.9	0.154	0.034	–7.6	–30.7	7.4	–38.0
$Cl_2Se{\cdots}F^-$	1.903	2.504	2.200	93.8	92.5	0.349	0.045	–7.3	–63.6	15.1	–78.6
$Cl_2Se{\cdots}Cl^-$	2.438	2.438	2.198	95.3	95.3	0.283	0.043	–5.8	–35.7	10.9	–46.6
$Cl_2Se{\cdots}Br^-$	2.600	2.438	2.198	96.0	96.0	0.283	0.043	–5.1	–32.1	10.7	–42.8
$Br_2Se{\cdots}F^-$	1.902	2.674	2.351	94.7	93.3	0.362	0.039	–7.0	–61.7	13.3	–75.0
$Br_2Se{\cdots}Cl^-$	2.440	2.601	2.353	96.4	96.3	0.289	0.041	–5.3	–34.5	9.4	–43.9
$Br_2Se{\cdots}Br^-$	2.602	2.602	2.353	97.1	97.1	0.290	0.041	–4.6	–31.2	9.3	–40.5
$F_2Te{\cdots}F^-$	2.054	2.054	1.930	84.2	84.2	0.162	0.038	–9.7	–72.4	7.9	–80.3
$F_2Te{\cdots}Cl^-$	2.608	2.026	1.927	85.2	86.3	0.134	0.035	–8.7	–42.5	5.9	–48.5
$F_2Te{\cdots}Br^-$	2.777	2.022	1.926	85.2	86.6	0.130	0.034	–8.7	–38.1	5.6	–43.7
$Cl_2Te{\cdots}F^-$	2.039	2.618	2.380	90.0	88.5	0.285	0.047	–8.2	–73.3	10.8	–84.1
$Cl_2Te{\cdots}Cl^-$	2.582	2.582	2.378	91.4	91.4	0.249	0.045	–6.8	–43.0	8.6	–51.6
$Cl_2Te{\cdots}Br^-$	2.745	2.579	2.378	91.4	91.8	0.246	0.045	–6.8	–38.6	8.4	–47.1
$Br_2Te{\cdots}F^-$	2.040	2.796	2.537	90.8	88.5	0.304	0.045	–8.3	–72.0	9.9	–81.9
$Br_2Te{\cdots}Cl^-$	2.582	2.750	2.533	92.5	92.0	0.258	0.041	–6.6	–42.0	7.6	–49.6
$Br_2Te{\cdots}Br^-$	2.751	2.751	2.535	92.8	92.8	0.259	0.043	–6.3	–37.7	7.6	–45.3

[a] Computed at ZORA-M06/QZ4P.

Table S2. Energy decomposition analyses (in kcal mol^{-1}) of D_2Ch•••A^- chalcogen bonds at the equilibrium geometries.[a]

D_2Ch•••A^-	ΔE_{int}	ΔV_{elstat}	ΔE_{Pauli}	ΔE_{oi}	ε(4a')	⟨4a'|np$_y$⟩	⟨2a'|np$_y$⟩	⟨2a''|np$_x$⟩	Pop$_{4a'}$	Pop$_{npy}$	$\Delta Q_{D_2Ch}^{VDD}$
F_2O•••F^-	–50.2	–55.3	87.5	–82.4	–6.4	0.12	0.13	0.07	0.48	1.61	–0.37
F_2O•••Cl^-	–37.9	–43.4	72.6	–67.1	–6.4	0.13	0.13	0.06	0.54	1.53	–0.35
F_2O•••Br^-	–56.5	–65.3	121.8	–113.0	–7.4	0.14	0.14	0.07	0.79	1.30	–0.48
Cl_2O•••F^-	–40.3	–38.8	79.1	–80.6	–5.7	0.11	0.14	0.04	0.52	1.59	–0.41
Cl_2O•••Cl^-	–31.0	–39.4	80.0	–71.5	–5.7	0.12	0.15	0.04	0.62	1.53	–0.41
Cl_2O•••Br^-	–57.5	–94.2	198.8	–162.1	–6.7	0.13	0.15	0.06	1.08	1.13	–0.62
Br_2O•••F^-	–17.4	–11.5	30.5	–36.4	–3.9	0.09	0.11	0.01	0.28	1.77	–0.26
Br_2O•••Cl^-	–7.6	–7.6	19.9	–19.9	–3.6	0.09	0.09	0.01	0.25	1.88	–0.19
Br_2O•••Br^-	–26.7	–41.5	89.0	–74.3	–5.1	0.13	0.16	0.04	0.68	1.51	–0.44
F_2S•••F^-	–66.3	–126.5	168.3	–108.1	–3.6	0.18	0.16	0.15	0.33	1.71	–0.35
F_2S•••Cl^-	–31.7	–56.8	74.5	–49.3	–3.1	0.22	0.16	0.11	0.31	1.73	–0.21
F_2S•••Br^-	–26.9	–47.2	60.7	–40.4	–2.9	0.22	0.16	0.10	0.29	1.76	–0.19
Cl_2S•••F^-	–77.5	–149.7	222.8	–150.7	–4.7	0.17	0.17	0.14	0.47	1.65	–0.47
Cl_2S•••Cl^-	–40.1	–72.9	109.9	–77.1	–4.1	0.20	0.20	0.10	0.46	1.63	–0.34
Cl_2S•••Br^-	–36.9	–64.2	95.4	–68.1	–4.1	0.20	0.19	0.10	0.47	1.65	–0.34
Br_2S•••F^-	–73.9	–148.4	234.2	–159.7	–4.6	0.16	0.14	0.18	0.52	1.64	–0.51
Br_2S•••Cl^-	–36.8	–69.7	110.9	–78.0	–4.0	0.19	0.20	0.10	0.48	1.64	–0.36
Br_2S•••Br^-	–34.6	–62.2	97.5	–69.8	–4.1	0.19	0.19	0.09	0.49	1.63	–0.36
F_2Se•••F^-	–73.7	–124.6	136.6	–85.7	–3.8	0.18	0.15	0.15	0.27	1.73	–0.32
F_2Se•••Cl^-	–42.3	–69.7	79.4	–52.0	–3.5	0.21	0.15	0.12	0.28	1.76	–0.23
F_2Se•••Br^-	–38.0	–62.1	71.3	–47.2	–3.5	0.22	0.16	0.12	0.29	1.75	–0.22
Cl_2Se•••F^-	–78.6	–132.4	160.1	–106.3	–4.3	0.18	0.16	0.13	0.36	1.69	–0.40
Cl_2Se•••Cl^-	–46.6	–78.2	99.2	–67.7	–4.0	0.22	0.18	0.11	0.38	1.69	–0.31
Cl_2Se•••Br^-	–42.8	–70.0	88.6	–61.5	–4.0	0.22	0.18	0.11	0.40	1.68	–0.32
Br_2Se•••F^-	–75.0	–129.4	166.0	–111.6	–4.2	0.17	0.17	0.13	0.39	1.69	–0.43
Br_2Se•••Cl^-	–43.9	–75.9	101.5	–69.5	–3.9	0.21	0.19	0.11	0.39	1.70	–0.33
Br_2Se•••Br^-	–40.5	–68.1	90.6	–62.9	–3.9	0.22	0.19	0.10	0.42	1.66	–0.34
F_2Te•••F^-	–80.3	–134.6	132.8	–78.6	–3.8	0.19	0.13	0.15	0.21	1.79	–0.32
F_2Te•••Cl^-	–48.5	–80.5	85.1	–53.1	–3.7	0.22	0.15	0.14	0.25	1.78	–0.24
F_2Te•••Br^-	–43.7	–71.7	76.6	–48.6	–3.7	0.23	0.15	0.13	0.26	1.79	–0.24
Cl_2Te•••F^-	–84.1	–140.0	147.9	–92.1	–4.0	0.18	0.16	0.14	0.28	1.77	–0.39
Cl_2Te•••Cl^-	–51.6	–85.8	96.7	–62.5	–3.8	0.24	0.18	0.13	0.32	1.74	–0.30
Cl_2Te•••Br^-	–47.1	–77.8	88.4	–57.6	–3.8	0.24	0.18	0.13	0.33	1.75	–0.30
Br_2Te•••F^-	–81.9	–137.3	151.8	–96.4	–3.9	0.17	0.16	0.14	0.30	1.76	–0.41
Br_2Te•••Cl^-	–49.6	–84.7	99.8	–64.7	–3.7	0.23	0.19	0.13	0.34	1.75	–0.32
Br_2Te•••Br^-	–45.3	–75.7	88.9	–58.6	–3.7	0.24	0.19	0.12	0.35	1.74	–0.32

[a] Computed at ZORA-M06/QZ4P; ε(4a') = 4a' orbital energy of the prepared D_2Ch fragment (in eV); ⟨Φ|np⟩ = overlap between the Φ fragment molecular orbital of the D_2Ch fragment and one of the np orbitals of the halide A^- (see Figure 1 and Figure S1); Pop = Gross population (in electrons) of indicated orbital.

Table S3. Activation strain and energy decomposition analyses (in kcal mol^{-1}) of $D_mZ\cdots A^-$ hydrogen bonds, halogen bonds, and chalcogen bonds at the equilibrium geometries.[a]

$D_mZ\cdots A^-$	ΔE	ΔE_{strain}	ΔE_{int}	ΔV_{elstat}	ΔE_{Pauli}	ΔE_{oi}
**FH•••F⁻**	–45.8	22.1	–67.9	–76.2	72.9	–64.6
**FF•••F⁻**	–30.5	28.0	–58.5	–44.6	84.3	–98.1
**FCl•••F⁻**	–52.3	14.0	–66.4	–94.5	124.9	–96.7
**FBr•••F⁻**	–62.3	10.0	–72.3	–104.0	113.8	–82.0
**FI•••F⁻**	–70.3	6.9	–77.2	–116.9	115.3	–75.7
**$F_2O\cdots F^-$**	–21.9	28.3	–50.2	–55.3	87.5	–82.4
**$F_2S\cdots F^-$**	–50.1	16.2	–66.3	–126.5	168.3	–108.1
**$F_2Se\cdots F^-$**	–62.4	11.4	–73.7	–124.6	136.6	–85.7
**$F_2Te\cdots F^-$**	–72.4	7.9	–80.3	–134.6	132.8	–78.6
**FH•••F⁻ [b]**	–53.0	19.7	–72.8	–76.4	68.8	–65.1
**FF•••F⁻ [b]**	–51.5	23.5	–75.0	–41.0	73.2	–107.1
**FCl•••F⁻ [b]**	–64.5	11.9	–76.4	–85.5	107.2	–98.1
**FBr•••F⁻ [b]**	–70.9	8.6	–79.5	–92.3	98.8	–86.0
**FI•••F⁻ [b]**	–75.0	6.1	–81.1	–103.5	100.0	–77.7

[a] Computed at ZORA-M06/QZ4P. [b] Computed at ZORA-BP86/TZ2P (from: L. P. Wolters, F. M. Bickelhaupt, *ChemistryOpen* **2012**, *1*, 96–105).

Table S4. Cartesian coordinates and bonding energies (in kcal mol^{-1}) for all stationary points of DO$^{\cdot}$, D_2O and $D_2O{\cdots}A^-$ structures, computed at ZORA-M06/QZ4P.

FO$^{\cdot}$

E = -225.53

O	0.000000	0.000000	-0.669361
F	0.000000	0.000000	-1.996459

BrO$^{\cdot}$

E = -203.38

O	0.000000	0.000000	-0.716646
Br	0.000000	0.000000	-2.414753

F_2O

E = -327.36

O	0.000000	0.000000	0.530005
F	0.000000	1.081727	1.380193
F	0.000000	-1.081727	1.380193

Br_2O

E = -270.91

O	0.000000	0.000000	0.537961
Br	0.000000	1.525334	1.527724
Br	0.000000	-1.525334	1.527724

$F_2O{\cdots}F^-$

E = -486.09

O	0.000000	0.000000	-0.225602
F	0.000000	-1.768187	-0.461578
F	0.000000	0.000000	1.148768
F	0.000000	1.768187	-0.461578

$Br_2O{\cdots}F^-$

E = -420.61

O	0.215250	0.240159	0.000000
Br	0.116751	2.208640	0.000000
Br	1.991889	-0.177707	0.000000
F	-0.152422	-1.889933	0.000000

$F_2O{\cdots}Cl^-$

E = -453.49

O	-1.960681	-0.506458	0.000000
F	-2.665916	1.125482	0.000000
F	-3.131164	-1.243692	0.000000
Cl	-0.458907	-2.090440	0.000000

$Br_2O{\cdots}Cl^-$

E = -393.24

O	-0.330115	0.071002	0.000000
Br	-2.193595	-0.310786	0.000000
Br	0.683659	-1.462885	0.000000
Cl	1.792355	1.695622	0.000000

$F_2O{\cdots}Br^-$

E = -446.39

O	-1.922131	-0.581375	0.000000
F	-2.673857	1.226423	0.000000
F	-3.118653	-1.305927	0.000000
Br	-0.438839	-2.086542	0.000000

$Br_2O{\cdots}Br^-$

E = -384.58

O	0.000000	0.000000	-0.110204
Br	0.000000	-2.130591	-0.811719
Br	0.000000	0.000000	1.733434
Br	0.000000	2.130591	-0.811719

ClO$^{\cdot}$

E = -209.86

O	0.000000	0.000000	-0.686493
Cl	0.000000	0.000000	-2.245995

Cl_2O

E = -278.29

O	0.000000	0.000000	0.531718
Cl	0.000000	1.396254	1.468767
Cl	0.000000	-1.396254	1.468767

$Cl_2O{\cdots}F^-$

E = -431.06

O	0.281231	-0.042828	0.000000
Cl	0.024952	2.123240	0.000000
Cl	1.915619	-0.262427	0.000000
F	-0.259704	-1.799491	0.000000

$Cl_2O{\cdots}Cl^-$

E = -401.05

O	0.000000	0.000000	-0.126941
Cl	0.000000	-2.093334	-0.707535
Cl	0.000000	0.000000	1.541897
Cl	0.000000	2.093334	-0.707535

$Cl_2O{\cdots}Br^-$

E = -396.75

O	0.214422	-0.039560	0.000000
Cl	-2.051130	-1.228676	0.000000
Cl	-0.136844	1.613929	0.000000
Br	2.157424	-0.339232	0.000000

Table S5. Cartesian coordinates and bonding energies (in kcal mol^{-1}) for all stationary points of DS$^{\cdot}$, D_2S and D_2S•••A^- structures, computed at ZORA-M06/QZ4P.

FS$^{\cdot}$

E = -209.89

S	0.000000	0.000000	-0.361026
F	0.000000	0.000000	-1.952436

ClS$^{\cdot}$

E = -165.23

S	0.000000	0.000000	-0.304586
Cl	0.000000	0.000000	-2.281516

BrS$^{\cdot}$

E = -155.28

S	0.000000	0.000000	-0.311952
Br	0.000000	0.000000	-2.433620

F_2S

E = -365.96

S	0.000000	0.000000	0.321224
F	0.000000	1.198432	1.359442
F	0.000000	-1.198432	1.359442

Cl_2S

E = -262.78

S	0.000000	0.000000	0.244988
Cl	0.000000	1.582753	1.493643
Cl	0.000000	-1.582753	1.493643

Br_2S

E = -240.53

S	0.000000	0.000000	0.203191
Br	0.000000	1.715680	1.548002
Br	0.000000	-1.715680	1.548002

F_2S•••F^-

E = -552.87

S	0.000000	0.000000	-0.454718
F	0.000000	-1.810328	-0.360719
F	0.000000	0.000000	1.176226
F	0.000000	1.810328	-0.360719

Cl_2S•••F^-

E = -452.64

S	-0.012196	-0.152726	0.000000
Cl	0.081504	2.322126	0.000000
Cl	2.032902	-0.304473	0.000000
F	-0.231367	-1.887256	0.000000

Br_2S•••F^-

E = -429.00

S	-0.002406	-0.209063	0.000000
Br	-0.013064	2.457623	0.000000
Br	2.202993	-0.309005	0.000000
F	-0.199099	-1.940903	0.000000

F_2S•••Cl^-

E = -505.37

S	0.154985	-0.438348	0.000000
F	1.888345	-0.512288	0.000000
F	0.244045	1.176172	0.000000
Cl	-2.287375	-0.225538	0.000000

Cl_2S•••Cl^-

E = -404.34

S	0.000000	0.000000	-0.421408
Cl	0.000000	-2.332398	-0.602411
Cl	0.000000	0.000000	1.626215
Cl	0.000000	2.332398	-0.602411

Br_2S•••Cl^-

E = -381.48

S	-0.004704	-0.050252	0.000000
Br	0.012695	2.449828	0.000000
Br	2.190059	-0.280894	0.000000
Cl	-0.464936	-2.350258	0.000000

F_2S•••Br^-

E = -493.25

S	-0.013072	0.197344	0.000000
F	0.402319	1.867628	0.000000
F	1.552764	-0.195713	0.000000
Br	-0.597190	-2.384114	0.000000

Cl_2S•••Br^-

E = -392.69

S	-0.020240	0.033658	0.000000
Cl	0.126111	2.367228	0.000000
Cl	2.002957	-0.268067	0.000000
Br	-0.607431	-2.402544	0.000000

Br_2S•••Br^-

E = -370.28

S	0.000000	0.000000	-0.420150
Br	0.000000	-2.493478	-0.683763
Br	0.000000	0.000000	1.786988
Br	0.000000	2.493478	-0.683763

Table S6. Cartesian coordinates and bonding energies (in kcal mol^{-1}) for all stationary points of $DSe^{\bullet}$, D_2Se and $D_2Se\cdots A^-$ structures, computed at ZORA-M06/QZ4P.

$FSe^{\bullet}$

E = -204.07

Se	0.000000	0.000000	-0.157815
F	0.000000	0.000000	-1.896154

$ClSe^{\bullet}$

E = -158.33

Se	0.000000	0.000000	-0.130855
Cl	0.000000	0.000000	-2.260949

$BrSe^{\bullet}$

E = -147.95

Se	0.000000	0.000000	-0.143220
Br	0.000000	0.000000	-2.414967

F_2Se

E = -355.46

Se	0.000000	0.000000	0.178041
F	0.000000	1.288271	1.333007
F	0.000000	-1.288271	1.333007

Cl_2Se

E = -255.08

Se	0.000000	0.000000	0.130618
Cl	0.000000	1.664020	1.499414
Cl	0.000000	-1.664020	1.499414

Br_2Se

E = -232.45

Se	0.000000	0.000000	0.098748
Br	0.000000	1.792512	1.558774
Br	0.000000	-1.792512	1.558774

$F_2Se\cdots F^-$

E = -554.66

Se	0.000000	0.000000	-0.488185
F	0.000000	1.928585	-0.399480
F	0.000000	0.000000	1.284980
F	0.000000	-1.928585	-0.399480

$Cl_2Se\cdots F^-$

E = -455.43

Se	0.001729	-0.093928	0.000000
Cl	0.070099	2.409373	0.000000
Cl	2.192185	-0.299727	0.000000
F	-0.258254	-1.978647	0.000000

$Br_2Se\cdots F^-$

E = -430.96

Se	-0.018526	-0.141862	0.000000
Br	-0.035206	2.532480	0.000000
Br	2.325461	-0.318690	0.000000
F	-0.268871	-2.026860	0.000000

$F_2Se\cdots Cl^-$

E = -506.40

Se	-0.028702	0.127766	0.000000
F	0.354562	1.975965	0.000000
F	1.707769	-0.187626	0.000000
Cl	-0.455738	-2.330469	0.000000

$Cl_2Se\cdots Cl^-$

E = -407.09

Se	0.000000	0.000000	-0.436147
Cl	0.000000	-2.427787	-0.662885
Cl	0.000000	0.000000	1.761645
Cl	0.000000	2.427787	-0.662885

$Br_2Se\cdots Cl^-$

E = -383.23

Se	-0.009305	-0.058323	0.000000
Br	-0.023205	2.543114	0.000000
Br	2.330046	-0.309640	0.000000
Cl	-0.534505	-2.441246	0.000000

$F_2Se\cdots Br^-$

E = -493.65

Se	-0.007560	0.199946	0.000000
F	0.372238	2.044845	0.000000
F	1.728441	-0.115092	0.000000
Br	-0.477524	-2.417064	0.000000

$Cl_2Se\cdots Br^-$

E = -394.71

Se	0.011849	0.037380	0.000000
Cl	0.118163	2.472660	0.000000
Cl	2.185873	-0.285944	0.000000
Br	-0.639413	-2.480037	0.000000

$Br_2Se\cdots Br^-$

E = -371.10

Se	0.000000	0.000000	-0.426870
Br	0.000000	-2.581941	-0.749593
Br	0.000000	0.000000	1.926060
Br	0.000000	2.581941	-0.749593

Table S7. Cartesian coordinates and bonding energies (in kcal mol^{-1}) for all stationary points of $DTe^{\cdot}$, D_2Te and $D_2Te{\cdots}A^-$ structures, computed at ZORA-M06/QZ4P.

$FTe^{\cdot}$

E = -194.85

Te	0.000000	0.000000	-0.082970
F	0.000000	0.000000	-1.992669

$ClTe^{\cdot}$

E = -146.00

Te	0.000000	0.000000	-0.049934
Cl	0.000000	0.000000	-2.374499

$BrTe^{\cdot}$

E = -134.51

Te	0.000000	0.000000	-0.033247
Br	0.000000	0.000000	-2.505272

F_2Te

E = -353.53

Te	0.000000	0.000000	0.112362
F	0.000000	1.383740	1.403736
F	0.000000	-1.383740	1.403736

Cl_2Te

E = -248.88

Te	0.000000	0.000000	0.058191
Cl	0.000000	1.764130	1.584623
Cl	0.000000	-1.764130	1.584623

Br_2Te

E = -224.05

Te	0.000000	0.000000	0.006372
Br	0.000000	1.895027	1.623467
Br	0.000000	-1.895027	1.623467

$F_2Te{\cdots}F^-$

E = -562.73

Te	0.000000	0.000000	-0.585977
F	0.000000	-2.043502	-0.379180
F	0.000000	0.000000	1.344522
F	0.000000	2.043502	-0.379180

$Cl_2Te{\cdots}F^-$

E = -459.00

Te	-0.038833	-0.084277	0.000000
Cl	0.171076	2.525717	0.000000
Cl	2.333895	-0.274715	0.000000
F	-0.147397	-2.120384	0.000000

$Br_2Te{\cdots}F^-$

E = -432.82

Te	-0.093958	-0.151721	0.000000
Br	0.028670	2.641380	0.000000
Br	2.439128	-0.297153	0.000000
F	-0.157099	-2.190718	0.000000

$F_2Te{\cdots}Cl^-$

E = -512.34

Te	-0.007965	0.073711	0.000000
F	0.580756	2.012695	0.000000
F	1.875664	-0.330622	0.000000
Cl	-0.391484	-2.506060	0.000000

$Cl_2Te{\cdots}Cl^-$

E = -408.17

Te	0.000000	0.000000	-0.563145
Cl	0.000000	-2.581554	-0.624091
Cl	0.000000	0.000000	1.814660
Cl	0.000000	2.581554	-0.624091

$Br_2Te{\cdots}Cl^-$

E = -382.30

Te	-0.094558	-0.072110	0.000000
Br	0.048009	2.674460	0.000000
Br	2.427342	-0.311811	0.000000
Cl	-0.430093	-2.632252	0.000000

$F_2Te{\cdots}Br^-$

E = -499.08

Te	-0.009213	0.144233	0.000000
F	0.692295	2.040299	0.000000
F	1.846881	-0.370781	0.000000
Br	-0.593640	-2.570586	0.000000

$Cl_2Te{\cdots}Br^-$

E = -394.99

Te	-0.057623	0.069302	0.000000
Cl	0.299913	2.622984	0.000000
Cl	2.288800	-0.318406	0.000000
Br	-0.590156	-2.623585	0.000000

$Br_2Te{\cdots}Br^-$

E = -369.26

Te	0.000000	0.000000	-0.566102
Br	0.000000	-2.748121	-0.701183
Br	0.000000	0.000000	1.968533
Br	0.000000	2.748121	-0.701183

Table S8. Cartesian coordinates and bonding energies (in kcal mol^{-1}) for all stationary points of FH, FX, FH•••F^{-} and FX•••F^{-} structures, computed at ZORA-M06/QZ4P.

FH

E = -243.05

H	0.000000	0.000000	0.907277
F	0.000000	0.000000	-0.007277

FF

E = -161.74

F	0.000000	-0.000000	0.687436
F	0.000000	-0.000000	-0.687436

FCl

E = -159.32

Cl	0.000000	0.000000	-0.154542
F	0.000000	0.000000	-1.772584

FBr

E = -161.54

Br	0.000000	0.000000	-0.017455
F	0.000000	0.000000	-1.768916

FI

E = -163.18

I	0.000000	0.000000	0.038897
F	0.000000	0.000000	-1.865829

FH•••F^{-}

E = -425.69

H	0.000000	0.000000	0.904026
F	0.000000	0.000000	-0.233472
F	0.000000	0.000000	2.041157

FF•••F^{-}

E = -329.12

F	0.000000	0.000000	-0.040740
F	0.000000	0.000000	-1.762181
F	0.000000	0.000000	1.680701

FCl•••F^{-}

E = -348.48

Cl	0.000000	0.000000	-0.011423
F	0.000000	0.000000	-1.878802
F	0.000000	0.000000	1.855957

FBr•••F^{-}

E = -360.74

Br	0.000000	0.000000	0.027908
F	0.000000	0.000000	-1.938771
F	0.000000	0.000000	1.994587

FI•••F^{-}

E = -370.29

I	0.000000	0.000000	0.046909
F	0.000000	0.000000	-2.042193
F	0.000000	0.000000	2.136012
